# Whey Protein Supplementation and Type 2 Diabetes Mellitus Risk Factors: An Umbrella Systematic Review of Randomized Controlled Trials

**DOI:** 10.1016/j.cdnut.2023.102017

**Published:** 2023-10-14

**Authors:** Gavin Connolly, Yu Wang, Robert E. Bergia, Eric M. Davis, Adam W. Byers, Jason B. Reed, Wayne W. Campbell

**Affiliations:** 1 Department of Nutrition Science, Purdue University, West Lafayette, IN, United States; 2 Libraries and School of Information Studies, Purdue University, West Lafayette, IN, United States

**Keywords:** dairy protein, milk protein, dietary protein, cardiometabolic, metabolic health, metabolic disease, metabolic syndrome, cardiovascular

## Abstract

Emerging research suggests whey protein (WP) supplementation may modify type 2 diabetes mellitus (T2DM) risk factors, including glucose control. As systematic reviews and/or meta-analyses of randomized controlled trials (RCTs) gain importance in nutrition literature, we conducted an umbrella systematic review to chronicle published systematic reviews and/or meta-analyses of RCTs pertinent to WP supplementation and T2DM modifiable risk factors. This review was conducted in accordance with Preferred Reporting Items for Overviews of Reviews standards. Potentially eligible articles were identified via a systematic search of 5 electronic health research databases (PubMed, Cochrane Library, CINAHL [EBSCO], Scopus, and SPORTDiscus [EBSCO]). Included articles were assessed for quality using the “A MeaSurement Tool to Assess systematic Reviews 2” critical appraisal tool. Thirteen articles, representing 109 unique RCTs, of the 2205 identified articles met the inclusion criteria. Nine articles (69%) were deemed high quality, 2 (15%) moderate quality, and 2 (15%) low quality. Findings from this umbrella review of 13 systematic reviews, including 12 meta-analyses, suggest WP may lower hemoglobin A1c, homeostasis model assessment of insulin resistance, and fasting insulin in groups classified as overweight/obese or at risk for or with metabolic syndrome; blood triglycerides in groups classified as overweight/obese or at risk for or with metabolic syndrome; and blood pressure in groups classified as overweight/obese. WP did not differentially affect C-reactive protein, body weight, body mass index, or waist circumference, nor did it adversely affect any T2DM risk factors. Insufficient evidence precluded assessing the influence of WP on glucose control-related outcomes in groups classified at lower risk for T2DM. Information regarding WP dose, duration, or types was insufficient to draw conclusions. Collectively, evidence suggests WP supplementation may improve multiple clinical indicators of glucose control, along with triglycerides and blood pressure, in groups of adults at increased risk of developing T2DM.

## Introduction

Diet is a leading modifiable cause of poor health globally, with poor diets accounting for 10.9 million deaths (22% of all deaths among adults in 2017). Of these deaths, type 2 diabetes mellitus (T2DM) was the third leading cause of diet-related deaths [1]. As such, dietary components can positively or negatively influence the prevention and treatment of T2DM. While dairy foods are widely consumed [2,3], evidence on their risk for T2DM is mixed (e.g., negative [[4], [5], [6]], neutral [[7], [8], [9]], beneficial [[10], [11], [12], [13]]).

Whey protein (WP) is one component of dairy that may confer favorable effects on some T2DM risk factors. Observational studies that provide information on self-reported dietary intakes of dairy foods and associations with T2DM risk do not consider WP supplementation. Human research on WP supplementation has mostly been conducted using randomized controlled trials (RCTs), which can assess causality between a specific exposure and outcome [14]. Primary outcomes typically are indices of body weight control, whole body composition, and skeletal muscle mass and function. Emerging research—usually as secondary or exploratory outcomes—has included assessments of how WP influences various facets of glycemic control and risk factors for T2DM. WP ingestion may facilitate improvement in postprandial glycemic responses by slowing gastric emptying and stimulating insulin secretion [[15], [16], [17], [18]]. In addition, some evidence suggests bioactive compounds found in whey, such as immunoglobulins, glutamine, lactoferrin, and lactalbumin, may be beneficial for improving metabolic parameters [[19], [20], [21], [22]].

Given the potential for WP to influence T2DM risk factors and the increasing prevalence of T2DM worldwide—which has nearly quadrupled in the last 4 decades [23]—it is important to better understand the effects of WP on risk factors for T2DM. We originally planned to perform a scoping review, which would identify pertinent literature on the effects of WP on T2DM risk factors, without presenting the findings. However, upon identifying the pertinent articles, we transitioned to an umbrella review to present a synthesis of research findings. Therefore, the purpose of this umbrella systematic review is to chronicle experimental research pertinent to WP supplementation and T2DM risk factors in humans by searching the scientific literature for peer-reviewed systematic reviews and/or meta-analyses of RCTs. Prominent risk factors include fasting and postprandial blood glucose and insulin, hemoglobin A1c (HbA1c), HOMA-IR, body weight, BMI, waist circumference, C-reactive protein (CRP), and blood lipids and lipoproteins.

## Methods

### Protocol

The protocol for this umbrella systematic review was developed in accordance with PRIOR (Preferred Reporting Items for Overviews of Reviews) recommended guidelines for reporting the evidence reviewed for this article [24]. This umbrella systematic review included systematic reviews and/or meta-analyses of RCTs in humans. We identified systematic reviews based on the definition provided by Cochrane [25]. The original scoping review protocol was registered with Open Science Framework (https://osf.io/8scfw/) because PROSPERO does not accept scoping review registrations. The umbrella review itself was not registered with PROSPERO because we had already performed the literature search, and the protocol was already registered with Open Science Framework.

### Research objective

To identify and synthesize evidence from systematic reviews and/or meta-analyses of RCTs pertinent to WP supplementation and T2DM risk factors.

### Search strategy, article selection process, and data extraction

Throughout this umbrella systematic review, the term “article” refers to a publication identified via the search process. Potentially eligible articles were identified via a systematic search of 5 electronic health research databases (PubMed, Cochrane Library [reviews and trials], CINAHL [EBSCO], Scopus, and SPORTDiscus [EBSCO]) from inception to 27 January, 2023. The 5 search strategies (one for each database) were developed by a health sciences librarian (JBR) in collaboration with other review team members (full search strategies are presented in Supplementary Table 1). The search was first created in PubMed using a combination of MeSH terms and keywords searched in the title/abstract fields and were based on the population, intervention, comparison, outcome, and study design (PICOS) information provided in Table 1. The entire search strategy, including the hedge for systematic reviews and/or meta-analyses [26], was translated into the other databases using controlled terms and free text terms in Cochrane Library, CINAHL, and SPORTDiscus; because Scopus does not offer a controlled vocabulary, only the free text terms were used. The only other differences in the search involved the search hedge for systematic reviews and/or meta-analyses. The PubMed version [26] was used in Cochrane because it includes MeSH terms; however, a specific hedge created for CINAHL [27] was used in both CINAHL and SPORTDiscus because it was also accessed via the EBSCO platform. The CINAHL version was also used as the foundation for the Scopus version because both platforms allow for proximity searching. The initial search was conducted on 10 May, 2021 and updated on 27 January, 2023. Duplicates were identified and removed using the built-in duplication checker in Covidence [28]. There were 5 total reviewers (AWB, EMD, GC, REB, and YW). The title and abstract of the articles, and subsequently the full text of articles, were independently assessed using Covidence by 2 independent reviewers to determine eligibility. A sixth reviewer (WWC) was consulted if the 5 primary reviewers could not reach consensus on article inclusion or exclusion. The reference lists of articles included were manually searched for additional articles which may fulfill the inclusion criteria. The inclusion and exclusion criteria are presented in Table 2.

**Table 1:** PICOS criteria for inclusion and exclusion of articles

Parameter	Description
Population	Humans of all ages across the life course
Intervention	Groups consuming whey protein or higher amounts of whey protein
Comparison	Groups not consuming whey protein or groups consuming lower amounts of whey protein
Outcome	Changes in clinical measures of risk factors for type 2 diabetes mellitus
Study design	Systematic reviews and/or meta-analyses of randomized controlled trials

**Table 2:** Inclusion and exclusion criteria used for article selection

Included	Excluded
Systematic reviews and/or meta-analyses of RCTs investigating whey protein intake and T2DM risk factors Published in the English language Published up to May 2021 Study population: Humans with no age or sex restrictions Outcome variables 1. Risk factors for T2DM include: i. Fasting blood glucose ii. Postprandial blood glucose iii. Glucose tolerance: OGTT or MGTT iv. 24-h CGM v. HbA1c vi. Fasting blood insulin vii. Postprandial insulin viii. HOMA-IR ix. Fasting blood lipids and lipoproteins (total cholesterol, LDL-cholesterol, HDL-cholesterol, and triglycerides) x. CRP, hs-CRP xi. Blood pressures (SBP and DBP) xii. Body weight, BMI, waist circumference	Not a systematic review and/or meta-analysis of RCTS, e.g.: 1. RCTs 2. Observational studies (e.g. cohort, case-control studies) Not published in the English language Study objectives not on whey protein intake No outcome of risk factor(s) for T2DM Articles that could not be accessed after contacting the authors Gray literature

CGM, continuous glucose monitoring; CRP, C-reactive protein; DBP, diastolic blood pressure; HbA1c, hemoglobin A1c; HDL-cholesterol, high-density lipoprotein cholesterol; hs-CRP, high-sensitivity C-reactive protein; MGTT, meal glucose tolerance test; OGTT, oral glucose tolerance test; RCT, randomized controlled trial; SBP, systolic blood pressure; T2DM, type 2 diabetes mellitus.

**FIGURE 1:**
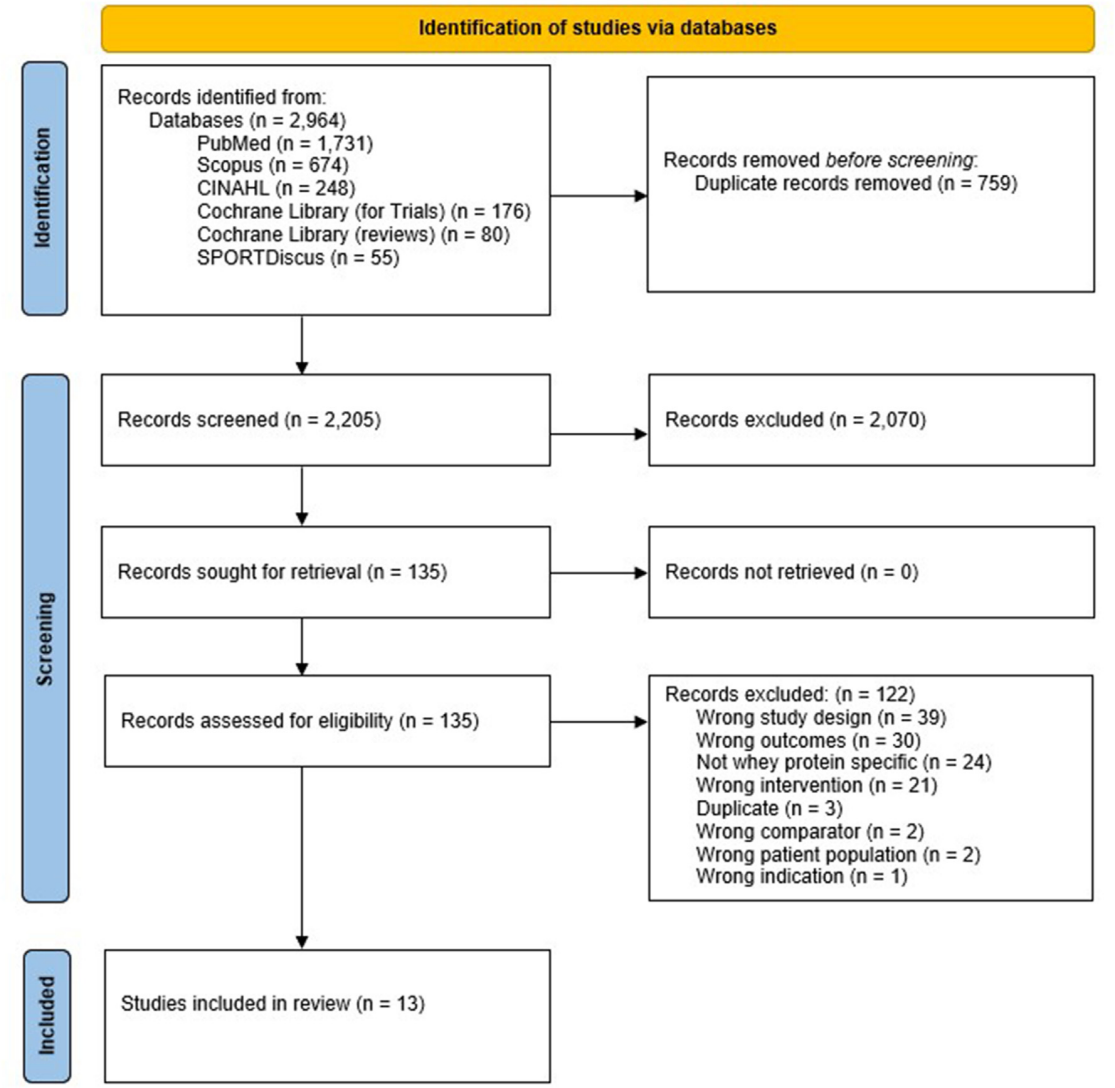
PRISMA flow diagram for this umbrella systematic review of randomized controlled trials assessing the effects of whey protein supplementation on type 2 diabetes mellitus risk factors.

The search process (Figure 1) and data extraction processe consisted of the following 3 stages: *1*) potential eligibility based on information provided in the title and abstract, *2*) confirmation of eligibility based on information provided in the purpose statement of the full text of qualified abstracts, and *3*) data extraction and critical appraisal of included systematic reviews and meta-analyses were assessed for methodological quality using AMSTAR 2 [29] from full-text articles if deemed qualified. The predetermined information extracted from all qualified full-text articles is shown in Supplementary Table 2.

The data extraction from each article and quality assessment of each article were performed by 2 independent reviewers using Covidence and crosschecked to ensure accuracy. Article authors were contacted for additional information if necessary. The authors of 3 articles were contacted regarding units of measure for outcomes, but we did not receive any responses.

This umbrella review is based on information contained in each of the systematic review articles. Other than documenting which original RCTs were included in each article (Supplementary Table 3), information contained in each RCT was not extracted or used for this umbrella review. We documented the method used by the authors of each article to assess risk of bias of primary RCTs (e.g., Jadad Scale, Cochrane Risk of Bias tool) included within each article and certainty of evidence for outcomes (e.g., Grading of Recommendations, Assessment, Development and Evaluations [GRADE]).

We also summarized and evaluated the data obtained from each article using a 3-step process: *1*) data are reported in Table 3, Table 4 and summarized by category and individual outcome in the Results; *2*) aggregated data among all articles are summarized in the Discussion; and *3*) the findings were evaluated and rationales for our interpretations described in the Discussion. Final evaluations with rationales include consideration of potential factors (e.g., health status, BMI classification, or physical activity level) contributing to interarticle heterogeneity or discrepancies.

**Table 3:** Characteristics, findings, and AMSTAR 2 quality ratings of included articles

Author	Population description	Dosage range of WP in RCTs	Duration range of included RCTs	Sex	Mean age range (y)	Participants BMI classifications	Included RCTs with exercise	Included RCTs with energy restriction	Findings	AMSTAR 2 Rating
Amirani et al. [19]	Aged ≥ 18 y with metabolic syndrome or conditions related to this syndrome	0.07–90 g/d	4–24 wk	F+M	Mean age range NR Age range of 18–77 y	Overweight or with obesity	N	Y	WP vs. controls: ↔ fasting BG ↓ fasting insulin ↓ HbA1c ↓ HOMA-IR ↓ total cholesterol ↓ LDL-C ↔ HDL-C ↓ TG	High
Badely et al. [30]	Overweight and obese participants aged ≥ 18 y	0.07–110 g/d	4–12 wk	F+M	23.474.3	Overweight or with obesity	Y	Y	WP vs. controls: ↓ Fasting BG ↓ SBP ↓ DBP ↓ HDL ↓ TG ↓ WC	Low
Bergia et al. [31]	Apparently healthy (not characterized as having a specific chronic disease), nonpregnant females with a mean age ≥ 19 y	6–48 g/d	6–52 wk	F	20–64.3	NR	Y	Y	WP vs. non-WP controls: ↔ body weight WP vs. CHO controls: ↔ body weight	High
Blair et al. [32]	“Apparently” healthy adults aged ≥ 35 y	0.04–60 g/d	2–78 wk	F+M	42–78	NR	Y	Y	The outcomes identified were body composition, bone mineral density, biochemical markers, such as blood glucose and lipids, and muscle strength and synthesis	High
Chiang et al. [33]	M/F adults with T2DM	30–75 g/d	Acute postprandial responses	F+M	54.9–65.5	Normal weight, overweight, and with obesity	NR	NA	WP vs. controls: ↓ BG at 60 and 120 min ↔ insulin at 60 and 120 min ↔ glucose iAUC ↑insulin iAUC	High
Kuo et al. [34]	Postmenopausal females	20–111 g/d	10–72 wk	F	54.9–74	Normal weight, overweight, or with obesity	Y	NR	WP vs. controls: ↔ body weight	High
Miller et al. [35]	Free-living adults aged ≥ 18 y	35–88 g/d	6–52 wk	F+M	Mean age range NR Age range of 18–72 y	Overweight, with obesity, or NR	Y	Y	WP as a dietary replacement vs. controls: ↔ body weight ↔ BMI ↔ WC WP as a dietary supplement vs. controls: ↔ body weight ↔ BMI ↔ WC Within-group MA: WP as a dietary replacement vs. controls: ↓ body weight ↔ BMI ↔ WC WP as a dietary supplement: ↔ body weight	High
Piri Damaghi et al. [36]	Healthy adults aged ≥ 18 y	22–56 g/d	2–36 wk	F+M	Mean age range NR Age range of 18–65 y	Normal weight, overweight, or with obesity	Y	NR	WP vs. controls: ↔ body weight	Low
Prokopidis et al. [37]	Mean age of ≥ 50 y	5–111 g/d	2–78 wk	F+M	50–80.8	Normal weight or overweight	NR	NR	WP vs. controls: ↔ CRP ↔ hs-CRP	High
Sepandi et al. [38]	Aged ≥ 18 y	15–111 g/d	4–72 wk	F+M	19–78	Normal weight, overweight, or with obesity	Y	NR	WP vs. controls: ↔ body weight ↓ BMI ↔ WC Within-group MA: ↔ body weight ↓ BMI ↓ WC	High
Wirunsawanya et al. [39]	Aged ≥ 18 y and were overweight or obese	20–75 g/d	2–65 wk	F+M	27–63	Overweight or with obesity	Y	Y	WP vs. controls: ↓ fasting BG ↓ body weight ↓SBP ↓DBP ↓ total cholesterol ↔ WC ↔ LDL-C ↑ HDL-C ↔ TG	Moderate
Zhang et al. [40]	F/M adults aged ≥ 18 y that were either healthy or disease carrying (metabolic syndrome, hypercholesterolemia, pre-HTN, and mild HTN) in all BMI categories	0.07–90 g/d	4–48 wk	F+M	23.7–54.5	Normal weight, overweight, or with obesity	Y	Y	WP vs. controls: ↓ TG ↔ total cholesterol ↔ LDL-C ↔ HDL-C	High
Zhou et al. [41]	F/M adults aged ≥ 18 y in any BMI category that were either healthy or have the following diseases: Pre-HTN, Mild HTN, COPD, and metabolic syndrome	0.07–60 g/d	4–36 wk	F+M	48.0–77.3	Overweight, with obesity, or NR	Y	NR	WP vs. controls: ↔ CRP	Moderate

AMSTAR 2, A MeaSurement Tool to Assess systematic Reviews; BG, blood glucose; COPD, chronic obstructive pulmonary disease; CRP, C-reactive protein; DBP, diastolic blood pressure; F, female; HbA1c, hemoglobin A1c; HDL-C, high-density lipoprotein cholesterol; hs-CRP, high-sensitivity C-reactive protein; HTN, hypertension; iAUC, incremental area under the curve; LDL-C, LDL cholesterol; M, male; MA, meta-analysis; N, no; NA, not applicable; NR, not reported; RCT, randomized controlled trial; SBP, systolic blood pressure; T2DM, type 2 diabetes mellitus; TG, triglycerides; WC, waist circumference; WP, whey protein; Y, yes.

**Table 4:** Results from meta-analyses of randomized controlled trials

Glycemic control outcomes		Lipid and lipoprotein outcomes		Body weight; BMI; WC		BPs; CRP; hs-CRP	
Fasting glucose	0.61 (−2.83, 1.62) [19] −1.42 (−1.52, −1.31)^1^ [30] 0.76 (0.14, 1.38)^1^ [39] NR [[31], [32], [33], [34], [35], [36], [37], [38],^40^,^41^]	Total cholesterol	−10.88 (−18.60, −3.17)^1^ [19] 0.53 (0.00, 1.06)^1^ [39] −0.11 mmol/L (−0.27, 0.05) [40] NR [[30], [31], [32], [33], [34], [35], [36], [37], [38],^41^]	Body weight	−0.12 kg (−0.90, 0.65) [31] −0.03 kg (−0.84, 0.78) [31] −4.20 kg (−7.67, −0.73)^1^ [35] 0.31 kg (−1.02, 1.63) [35] −1.85 kg (−6.73, 3.02) [35] 0.10 kg (−4.43, 4.62) [35] −0.46 kg (−1.92, 1.00) [36] 0.56 (0.30, 0.81)^1^ (39) −0.05 kg (−0.15, 0.05) [38] −0.79 kg (−1.61, 0.06) [38] −0.12 kg (−0.29, 0.06) [34] NR [19,30,32,33,37,40,41]	SBP	−7.46 (−9.39, −6.13)^1^ [30] NR [19,[31], [32], [33], [34], [35], [36], [37], [38], [39], [40], [41]]
Fasting insulin	−0.94 (−1.68, −0.21)^1^ [19] NR [30,[32], [33], [34],[36], [37], [38],^40^,^41^]	LDL-C	−8.47 (−16.59, −0.36)^1^ [19] 0.31 (−0.02, 0.63) [39] −0.08 mmol/L (−0.23, 0.07) [40] NR [[30], [31], [32], [33], [34], [35], [36], [37], [38],^41^]	BMI	−0.67 (−2.85, 1.51) [35] −0.16 (−0.31, 0.00)^1^ [38] −0.77 (−1.54, 0.00)^1^ [38] NR [19,[30], [31], [32], [33], [34],^36^,^37^,[39], [40], [41]]	DBP	−5.68 (−6.69, −4.67)^1^ [30] NR [19,[31], [32], [33], [34], [35], [36], [37], [38],^40^,^41^]
Postprandial glucose	At 60 min: −2.67 mmol/L (−3.62, −1.72)^1^ [33] At 120 min: −1.59 mmol/L (−2.91, −0.28)^1^ [33] iAUC: −360 mmol/L × min (−912, 48) [33] NR [19,30,32,34,[36], [37], [38],^40^,^41^]	HDL-C	−0.13 (−1.74, 1.48) [19] −6.07 (−7.53, −4.61) [30] 0.42 (0.05, 0.80)^1^ [39] 0.01 mmol/L (−0.04, 0.05) [40] NR [[31], [32], [33], [34], [35], [36], [37], [38],^41^]	WC	2.76 (−3.83, −1.69)^1^ [30] −0.92 cm (−4.86, 3.03) [35] 0.47 cm (−4.19, 5.14) [35] −0.86 cm (−3.76, 2.04) [35] 0.46 (−0.66, 1.57) [39] −0.45 cm (−0.86, −0.03)^1^ [38] −4.62 cm (−13.50, 4.27) [38] NR [19,31,[32], [33], [34],^36^,^37^,^40^,^41^]	CRP	−0.42 mg/L (−0.96, 0.13) [41] −0.09 mg/L (−0.39, 0.21) [37] NR [19,[30], [31], [32], [33], [34], [35], [36],[38], [39], [40]]
Postprandial insulin	At 60 min: 102 pmol/L (−110, 313) [33] At 120 min: 120 pmol/L (−91, 332) [33] iAUC: 24.7 nmol/L × min (1.7, 47.6) [33] NR [19,[30], [31], [32],[34], [35], [36], [37], [38], [39], [40], [41]]					hs-CRP	−0.32 mg/L (−0.99, 0.35) [41] 0.12 mg/L (−0.42, 0.66) [37] NR [19,[30], [31], [32], [33], [34], [35], [36],[38], [39], [40]]
24-h CGM iAUC	NR [19,[30], [31], [32], [33], [34], [35], [36], [37],[39], [40], [41]]	TG	−17.12 (−26.52, −7.72)^1^ [19] −18.9 (−22.49, −15.30)^1^ [30] −0.42 (−0.79, −0.06) [39] 0.11 mmol/L (−0.21, 0)^1^ [40] NR [[31], [32], [33], [34], [35], [36], [37], [38],^41^]				
HbA1c	−0.15 (−0.29, −0.01)^1^ [19] NR [[30], [31], [32], [33], [34], [35], [36], [37], [38], [39], [40], [41]]						
HOMA-IR	−0.20 (−0.36, −0.05)^1^ [19] NR [[30], [31], [32], [33], [34], [35], [36], [37], [38], [39], [40], [41]]						

Values are weight mean differences and 95% confidence intervals (CIs).

Abbreviations: BP, blood pressure; CGM, continuous glucose monitoring; CRP, C-reactive protein; DBP, diastolic blood pressure; HbA1c, hemoglobin A1c; HDL-C, high-density lipoprotein cholesterol; hs-CRP, high-sensitivity C-reactive protein; iAUC, incremental areas under the curve; LDL-C, low-density lipoprotein cholesterol; NR, not reported; SBP, systolic blood pressure; TG, triglycerides; WC, waist circumference.

1 Indicates a significant difference between groups or between baseline and postintervention values (*P* < 0.05);

## Results

### Study features and participant characteristics

The search identified 2964 articles. After removing duplicates (*n* = 759), a total of 2205 articles remained. The titles and abstracts of the 2205 articles, and subsequently the full texts of 135 articles, were independently assessed using Covidence by 2 independent reviewers to determine eligibility. Thirteen articles meeting the inclusion criteria were identified (Figure 1). The characteristics of these articles are provided in Table 3. Of the 13 articles, 12 were systematic reviews and meta-analyses combined [19,[30], [31], [32], [33], [34], [35], [36], [37], [38], [39], [40]] and one was a systematic review [32]. Among the 13 articles, the number of RCTs per article ranged from 5 [33] to 37 [30], which cumulatively represents 109 unique RCTs (accounting for duplication; Supplementary Table 3). The primary outcomes reported included: indices of glycemic control [19,30,33,39]; blood lipids and lipoproteins [19,30,39,40]; blood pressures [30,39]; body weight [31,34,36,38,39]; BMI [35,38]; waist circumference [30,35,38,39]; and CRP and high-sensitivity CRP (hs-CRP) [37,41]. For an outcome that had more than one article report on it, the amount of overlap among and between studies is provided in Supplementary Tables 4–12. Eleven articles included both males and females [19,30,32,33,[35], [36], [37], [38],[39], [40], [41]], and 2 included only females [31,34]. Among the 13 articles, the total number of participants ranged from 134 [33] to 2344 [30], and the age of participants ranged from 18 [19,30,35,36] to 81 [30] y. Participants were classified, based on BMI, as overweight or with obesity in 6 articles [19,30,35,37,39,41]; normal weight, overweight, or with obesity in 4 articles [33,36,38,40]; normal weight and overweight in one article [34]; and not classified in 2 articles [31,32]. Intervention durations ranged from acute postprandial responses [33] to 78 wk [32,37]. Whey protein supplementation doses in the intervention groups ranged from 0.04 g/d [32] to 111 g/d [37,38]. Ten articles included RCTs with exercise components [30,32,34,36,[38], [39], [40], [41]]; 1 article excluded RCTs with exercise components [19]; and 2 articles did not report on exercise [33,37]. Seven articles included RCTs that had an energy restriction component [19,30,32,35,39,40], while 4 articles did not report on energy intakes [33,36,38,41]. Five articles included RCTs with both exercise and energy restriction [31,32,35,39,40].

### Quality of selected studies

Based on the AMSTAR 2 critical appraisal tool, 9 articles (69%) were deemed high quality [19,[31], [32], [33], [34], [35],^37^,^38^,^40^], 2 articles (15%) were moderate quality [39,41], and 2 articles (15%) were low quality [30,36] (Table 3). Supplementary Table 13.1presents the AMSTAR 2 assessment for each question and overall rating for each included article. Additionally, our review meets the AMSTAR 2 criteria to be deemed high quality (Supplementary Table 13.2). The primary reasons for a moderate or low-quality score were the authors *1*) did not consider risk of bias when interpreting the results of the review [30,36]; *2*) did not report on the sources of funding for the studies included in the review [30,36,38,39,41]; or *3*) did not report any potential sources of conflict of interest, including any funding they received for conducting the review [30,36,41].

### Results from included articles

Results from the 13 articles are provided in Table 4, and the results from subgroup analyses are provided in Supplementary Table 14. All values are weighted mean differences (WMDs) in change values between WP and comparator interventions unless otherwise stated. The WMD values do not identify the direction of any intervention-related changes in outcomes of interest, unless noted. Units of measure for outcomes are included if they were reported in the original articles. Three articles did not provide units of measures [19,30,39].

### Comparator interventions

Assessment of WP compared to various specific comparator interventions is beyond the scope of this umbrella review because 11 of the 13 articles did not do subgroup analyses based on specific types of comparators.

### Glycemic control

Two articles reported a reduction [30,39], and 1 article reported no difference [19] in fasting blood glucose with WP compared with comparator interventions. One article reported a reduction in fasting insulin with WP compared with comparator interventions [19]. One article reported reductions in postprandial glucose and no differences in postprandial insulin at 60 and 120 min with WP compared with comparators [33]. One article reported no difference in glucose incremental area under the curve (iAUC) and greater insulin iAUC with WP compared with comparators [33]. One article reported reductions in HbA1c and HOMA-IR with WP compared with comparator interventions [19].

### Blood lipids and lipoproteins

Two articles reported a reduction [19,39], and 1 article reported no difference [40] in total cholesterol with WP compared with comparator interventions. One article reported a reduction [19], and 2 articles reported no difference [39,40] in LDL-cholesterol with WP compared with comparator interventions. One article reported a reduction [30], 1 article reported a difference favoring [39], and 2 articles reported no difference [19,40] in HDL-cholesterol with WP compared with comparator interventions. Three articles reported a reduction [19,30,40], and 1 article reported no difference [39] in triglycerides with WP compared with comparator interventions.

Subgroup analyses by Zhang et al. [40] identified WP reduced triglycerides for participants with a BMI ≥30 kg/m^2^ but not <30 kg/m^2^. Subgroup analyses also identified WP reduced triglycerides when the dose was ≥30 g/d but not <30 g/d, and when participants were not exercise training or in energy restriction. Whey protein did not influence triglycerides when participantes were exercise training or in energy restriction. Whey protein did not influence triglycerides in participants with a baseline LDL-cholesterol of ≥3.2 mmol/L or <3.2 mmol/L [40].

### Blood pressures

Two articles reported a reduction in both systolic and diastolic blood pressures with WP compared with comparator interventions [30,39].

### C-reactive protein

Two articles reported no difference in CRP or hs-CRP with WP compared with comparator interventions [37,41].

Subgroup analyses by Zhou et al. [41] identified WP reduced CRP when the dose was ≥20 g/d but not <20g/d. In addition, WP reduced CRP in participants with a baseline CRP ≥3 mg/dL but not <3 mg/dL. WP did not influence CRP independent of intervention durations <12 wk or ≥12 wk [41].

Subgroup analyses by Prokopidis et al. [37] identified that WP reduced CRP when the intervention duration was ≤8 wk but not >8 wk and in participants with BMI <25 kg/m^2^ but not with BMI ≥25 kg/m^2^. Additionally, WP did not influence CRP in individuals aged <60 or ≥60 y; with sarcopenia or prefrailty; or when the dose was <30 g/d or ≥30 g/d [37].

### Body weight, BMI, and waist circumference

Among 6 articles, 5 reported no differential changes in body weight for WP compared with comparator interventions [31,34,36,38], WP compared with carbohydrates [31,35], and WP compared with other protein sources [35]. One article reported a reduction in body weight for WP compared with comparator interventions [39]. Based on pre- to postintervention assessments (within-group meta-analysis), 2 articles reported no change in body weight with WP as a dietary supplement [35,38], while 1 article [35] reported WP reduced body weight when used as a dietary replacement.

One article reported a reduction in BMI with WP from pre- to postintervention and WP compared with comparators [38], while 1 article reported no difference in BMI for WP compared with carbohydrate interventions [35].

One article reported a reduction [30] and 3 articles reported no difference [35,38,39] in waist circumference with WP compared with comparator interventions. In addition, 2 articles [35,38] reported no difference in waist circumference with WP from pre- to postintervention.

Subgroup analyses by Bergia et al. (31), Miller et al. [35], and Kuo et al. [34] identified WP did not influence changes in body weight with or without resistance training. In addition, the type of WP, isolate or concentrate, did not influence body weight [29]. Similarly, subgroup analyses by Piri Damaghi et al. [36] indicated WP did not influence body weight, independent of dose, intervention duration, or participant sex. However, WP reduced body weight in participants classified as overweight or with obesity, but not normal weight; independent of weight classification, WP reduced body weight without, but not with, exercise [36].

## Discussion

To the authors’ knowledge, this is the first umbrella systematic review to search for, chronicle, and assess the quality of peer-reviewed published scientific literature of systematic reviews and/or meta-analyses of RCTs pertinent to WP supplementation and T2DM risk factors. Among the 13 articles included in this umbrella review, evidence indicates that WP may improve measures of glucose control in individuals classified as overweight or with obesity [19,30,39] or at risk for or with metabolic syndrome [19]; triglycerides in individuals classified as overweight or with obesity [19,30,40] or at risk for or with metabolic syndrome [19,30,42]; and blood pressure in individuals classified as overweight or with obesity [30,39]. WP supplementation did not differentially affect other blood lipids and lipoproteins, CRP, hs-CRP, body weight, BMI, or waist circumference. None of the 13 articles identified any negative responses of WP on T2DM-related outcomes of interest.

Critical appraisal of this research includes reviewing the quality of the current umbrella review; the quality of each systematic review and/or meta-analysis included in this umbrella review; and the quality of individual RCTs included in each systematic review and/or meta-analysis. AMSTAR 2 critically appraises the conduct of included systematic reviews of RCTs. Although “study quality” is not well defined, it relates to how well a study is designed, conducted, analyzed, and reported [43]. Based on AMSTAR 2, the methodological quality of the 13 articles varied from high (69%; 9 articles) [19,[31], [32], [33], [34], [35],^37^,^38^,^40^] to moderate (15%; 2 articles) [39,41], to low (15%; 2 articles) [30,36] among reviews. The primary reasons for a moderate or low-quality score were the authors *1*) did not consider risk of bias when interpreting the results of the review [30,36]; *2*) did not report on the sources of funding for the studies included in the review [30,36,38,39,41]; or *3*) did not report any potential sources of conflict of interest, including any funding they received for conducting the review [30,36,41]. The authors of AMSTAR 2 state that although they “believe seven domains can critically affect the validity of a review and its conclusions,” they “recognize that the items listed as critical domains will not always be regarded as critical” [29]. As such, with AMSTAR 2, if a systematic review receives a lower rating, it does not mean that it inherently impacts the quality of research, as this is more relevant to the individual RCTs included within each review.

To independently assess the methodological quality of RCTs, a tool such as the Jadad Scale [44]—the most widely used such assessment tool [45]—is required, which was used in 2 (15%) articles [40,41]. The Jadad Scale assesses the methodological quality of an RCT based on 5 questions pertaining to 3 key features, specifically randomization, masking, and accountability of all patients, including withdrawals [44]. However, it does not account for allocation concealment, which is considered to be essential to avoid bias by The Cochrane Collaboration [43]. As such, to more comprehensively assess risk of bias of each individual RCT included in a systematic review, the Cochrane Risk of Bias tool was developed and is often used, which was used in 9 (69%) articles [19,30,[31], [32], [33], [34],^36^,^37^,^39^]. An in-depth discussion on the development, use, and strengths and limitations of each quality assessment tool mentioned above is beyond the scope of this review, and further information on each can be found in the following references [29,[43], [44], [45], [46], [47]]. The information from these assessments of these articles is provided within the original publications. Our results should be interpreted with this in mind because a high-quality systematic review included in our review could have potentially included low-quality empirical studies.

Importantly, AMSTAR 2, the Jadad Scale, or Cochrane Risk of Bias does not rate the certainty of evidence for outcomes based on included RCTs, which can be used for making clinical practice recommendations. To rate the certainty of evidence in systematic reviews for making clinical practice recommendations, an approach such as the GRADE approach is required, which provides an overall rating to the body of evidence of included RCTs for an outcome [[47], [48], [49]]. However, only 4 (31%) of 13 included articles [19,33,[34], [35], [36], [37]] used GRADE for determining the certainty of evidence for the outcomes assessed. The reports of GRADE assessments for the outcomes included in these articles are provided in Supplementary Table 15.

## Glycemic control

### Biological plausibility.

WP is a rich source of amino acids, and bioactive substances such as immunoglobulins, glutamine, lactoferrin, and lactalbumin, which augment glycemic control via several interconnected mechanisms [[19], [20], [21], [22]]. These mechanisms include amino acid-induced insulin secretion, stimulating the secretion of incretin hormones, inhibiting dipeptidyl peptidase 4, and a slower rate of gastric emptying [50].

Amino acids, in particular, the branched-chain amino acids leucine, isoleucine, and valine, have strong insulinotropic effects, with leucine being the most potent [51,52]. Leucine stimulates glutamate dehydrogenase activity in the β cells of the pancreas, which results in an increase in Krebs cycle activity and oxygen consumption in the β cells and a subsequent increase in insulin secretion [51]. WP consumption also stimulates the secretion of the gut-derived incretin hormones glucagon like peptide-1 (GLP-1) and gastric inhibitory polypeptide (GIP), which have strong insulinotropic effects [15,16,18,41]. GLP-1 and GIP act via G protein-coupled receptors that are highly expressed in β cells of the pancreas to stimulate β cell activity and insulin secretion [15,16,18,33,53]. In addition to incretins stimulating insulin secretion, they stimulate β cell proliferation and reduce apoptosis of β cells [53]. Whey peptides also inhibit dipeptidyl peptidase 4, a protein that increases the degradation of GIP and GLP-1, thereby improving blood glucose control [15,16,18]. A slower rate of gastric emptying also improves blood glucose control [16,48,49]. An increase in GLP-1 also slows the rate of gastric emptying mediated through afferent signals from the vagus nerve to the brain. Additionally, WP consumption stimulates the secretion of the gut-derived hormones cholecystokinin (CCK) and peptide YY (PYY), which also acts to slow the rate of gastric emptying mediated through central-related mechanisms [16,54,55].

### Evidence from the systematic reviews of RCTs.

Overall, evidence regarding glycemic control suggests a favorable effect with WP compared with comparator interventions in individuals classified as overweight or with obesity or at risk for or with metabolic syndrome.

Among the 3 articles that reported on fasting blood glucose, 2 reported a decrease in fasting blood glucose with WP compared with comparator interventions [30,39], while 1 article found no difference between interventions [19]. As 3 (75%) of 4 RCTs included in Wirunsawanya et al. [39] were also included in Badely et al. [30], we are only comparing the articles by Amirani et al. [19] and Badely et al. [30] that may factor into why differences were observed between articles for fasting blood glucose. Although both articles included participants who were overweight or obese, participants in Badely et al. [30] were otherwise healthy with no diagnosed diseases, whereas in Amirani et al. [19], participants had metabolic syndrome or conditions related to metabolic syndrome. In addition to differences in health status, Amirani et al. [19] excluded RCTs with an exercise component, whereas Badely et al. [30] included RCTs with an exercise component. The participant characteristics in Amirani et al. [19] indicated they were more metabolically dysregulated than the participants in Badely et al. [30]. It may be that interventions with WP alone in the participant population in Amirani et al. [19] were not sufficient to induce a benefit compared with comparator interventions, whereas in the participant population in Badely et al. [30], it was, in addition to including RCTs with an exercise component, which is a potent stimulus for improving blood glucose control. The differences in health status and exercise likely factor into why differences between articles were observed. However, Amirani et al. [19] did report a low quality of evidence for fasting blood glucose based on the GRADE approach.

The one article by Amirani et al. [19] that reported on other measures of glycemic control showed that in adults who were overweight or with obesity and with metabolic syndrome or conditions related to metabolic syndrome, there were reductions in fasting insulin, HOMA-IR, and HbA1c with WP compared with comparator interventions. Based on GRADE, the certainties of evidence were moderate for insulin and HbA1c and low for HOMA-IR.

The one article by Chiang et al. [33] that reported on postprandial glucose and insulin showed reductions in glucose, but not insulin, at 60 and 120 min. Additionally, glucose iAUC was not different, but insulin iAUC was greater with WP compared with comparator interventions. These findings should be interpreted with caution as the number of RCTs in the meta-analyses for these outcomes ranged from 2 to 4, with moderate to high heterogeneities, and the certainties of evidence based on GRADE were deemed very low to low.

Taken together, the evidence from mechanistic studies and from included systematic reviews and meta-analyses of RCTs suggest that WP may improve measures of glycemic control in groups classified as overweight or with obesity or at risk for or with metabolic syndrome. The low to very low certainty of evidence based on GRADE assessments underscores caution regarding the effectiveness of WP in improving measures of glycemic control. Insufficient evidence precluded assessing the influence of WP on glucose control-related outcomes in groups classified with normal body weight and at lower risk for impaired glucose control or T2DM.

## Blood lipids and lipoproteins

### Biological plausibility

There are several biologically plausible mechanisms by which WP may improve lipid metabolism and lipid profiles. WP consumption influences lipid metabolism by stimulating lipoprotein lipase, promoting hepatic lipid metabolism, inhibiting intestinal fatty acid and cholesterol absorption, and increasing the excretion of fecal sterols mediated by the presence of bioactive compounds such as β-lactoglobulin and sphingolipids in WP, and the downregulation of the expression of genes related to cholesterol metabolism, lipogenesis, and fatty acid transporters [[56], [57], [58]].

### Evidence from the systematic reviews of RCTs

Overall, the evidence regarding blood lipids and lipoproteins indicates either a favorable or neutral effect with WP compared with interventions in adults who were classified as overweight or moderately obese, without [27,39,40] or with metabolic syndrome or conditions related to metabolic syndrome [19,40]. Only 1 RCT included in Zhang et al. [40] studied participants who were normal weight, thereby precluding us from drawing conclusions on the effects of WP on lipids and lipoproteins in individuals with normal weight. Regarding triglycerides, the 3 articles that reported favorable effects of WP included 28 [30], 18 [19], and 13 [40] RCTs. In comparison, the article reporting a neutral effect of WP contained only 6 RCTs. As such, the favorable effects of WP on triglycerides may, in part, relate to the larger sample sizes and improved power to detect an effect. Based on the Jadad Scale and risk of biases, the majority of RCTs with assessments of lipids and lipoproteins were of moderate to high quality, with low risk of biases. Yet, based on GRADE the certainty of evidence for the outcomes assessed was deemed low to very low. Collectively, results from these assessment tools underscore caution regarding the effectiveness of WP in improving blood lipids and lipoproteins.

## Blood pressures

### Biological plausibility

Hypertension is a modifiable risk factor associated with T2DM and mortality. Elevated blood pressure is, in part, reflective of underlying insulin resistance of the vasculature and kidney [59]. Also, dysregulations in carbohydrate metabolism are more common in individuals with hypertension [60,61], indicating a bidirectional pathogenic relationship between T2DM and hypertension [62]. Reductions in blood pressures observed with WP, may in part, be explained by the presence of angiotensin-converting enzyme-inhibitory peptides found in WP, which decreases the enzymatic conversion of angiotensin I to vasoconstrictor angiotensin II and augments nitric oxide-mediated vasodilation [[63], [64], [65]].

### Evidence from the systematic review of RCTs

The 2 articles reporting on blood pressures showed WP reduced systolic and diastolic blood pressures in adults classified as overweight or with obesity [30,39]. These findings should be interpreted with caution because the articles were deemed low [30] and moderate [39] quality (AMSTAR 2), and one [30] did not report on their Cochrane Risk of Bias assessments.

## C-reactive protein

### Biological plausibility

Increased incidence of T2DM is associated with increased CRP (a marker of inflammation), increased oxidative stress, and a decreased concentration of the antioxidant glutathione [66]. Glutathione exists in 2 states, reduced glutathione (GSH) and oxidized glutathione (GSSG). The ratio of GSH to GSSG is a measure of oxidative stress, with a decreased ratio of GSH to GSSG indicating increased oxidative stress and increased inflammation. WP can exert antioxidant and anti-inflammatory effects by increasing the synthesis of the GSH, resulting in reductions in measures of inflammation including CRP and insulin resistance [[67], [68], [69]].

### Evidence from the systematic reviews of RCTs

The 2 articles reporting on CRP and hs-CRP found no differential effects with WP compared with comparator interventions in their overall analyses [37,41]. The articles were deemed moderate [41] and high quality [37] (AMSTAR 2), and the included RCTs had moderate to high Jadad scores [41] with moderate and high certainty of evidence for CRP and hs-CRP, respectively [37]. The authors noted their reviews were limited by considerable heterogeneity among studies [37,41], making interpretation of findings complicated. Subgroup analyses by Zhou et al. [41] identified baseline CRP as a potential effect modifier: a reduction in CRP was observed in participants with a baseline CRP ≥3 mg/L but not <3 mg/L. This is important because a CRP ≥3 mg/L is associated with greater T2DM incidence [63], and an intervention with WP may be more effective in individuals at greater risk of developing T2DM.

## Body weight, BMI, and waist circumference

### Biological plausibility

WP may mediate an improvement in body weight, BMI, and waist circumference by altering ingestive behaviors. The consumption of WP results in an increase in insulin, which is linked with increased satiety and a decrease in the orexigenic hormone ghrelin [70]. WP consumption also stimulates the secretion of the gut-derived hormones GLP-1, CCK, and PYY, which increase satiety and slow gastric emptying mediated through afferent signals from the vagus nerve to the brain [15,17,18,50]. WP consumption may also influence ingestive behaviors mediated through hypothalamic neuropeptides. Energy intake may be decreased due to WP-induced increases in the anorexigenic neuropeptide pro-opiomelanocortin and decreases in the orexigenic neuropeptides agouti-related peptide and neuropeptide Y [71]. In addition, dietary protein consumption, inclusive of WP, relative to carbohydrate and fat augments energy expenditure by resulting in a 15% to 20% higher thermic effect of feeding, or diet-induced thermogenesis [[72], [73], [74], [75]]. However, research associating these acute or short-term dietary responses with longer-term changes in body weight and body composition is inconsistent [[76], [77], [78]].

### Evidence from the systematic reviews of RCTs

Among adults who were healthy [31,36], normal weight [36], overweight [35,36,39], with obesity [35,36,39], hyperlipidemic [35], or postmenopausal [34], evidence regarding WP effects on body weight, BMI, and waist circumference are predominantly neutral [31,34,35,36,38,39], with some positive effects [30,35,38]. Risk of bias assessments of included RCTs varied. The discordance in findings may stem from articles differing in experimental features including amounts or types of physical activity/exercise and energy intake relative to energy expenditure.

Miller et al. [35] reported WP reduced body weight when used as a dietary replacement for other sources of energy, with 3 of the 4 RCTs conducted with participants not performing resistance training. In contrast, WP did not differentially affect body weight when used as a dietary supplement providing additional energy intake, with 7 of the 9 RCTs including resistance training. These results suggest consuming WP as a dietary replacement without resistance training, compared to WP as a dietary supplement with resistance training, may more effectively promote weight loss. However, this interpretation should be made with caution since relative changes in body composition (fat and fat-free masses) are not considered. Independent of resistance training, subgroup analyses by Bergia et al. [31], Miller et al. [35], and Kuo et al. [34] indicated WP did not influence changes in body weight.

## Dose-response

The dose of WP is an important factor to consider as there may be minimum and maximum thresholds of the amount of WP and its bioactive components required to elicit beneficial effects on T2DM risk factors. Additionally, the dose that is beneficial for one or more outcomes may not be the same for all risk factors for T2DM. However, among the articles included in this review, none reported responses to 3 or more doses of WP (i.e., a dose-response) for any risk factor for T2DM. Given the wide range of WP doses among individual RCTs included within each article in this review, this likely factors into the magnitude and directionality of the findings of the meta-analyses from each article. For example, it is unlikely that WP doses as low as 0.04 g/d or 0.07 g/d will elicit the same responses as doses as high as 111 g/d. Collectively, new research focused on dose-responses to acutely and chronically consuming whey protein on T2DM risk factors seem warranted.

## Strengths and limitations

The strengths of this umbrella systematic review include that, to the best of the authors’ knowledge, this is the first umbrella systematic review to search for, chronicle, and assess the quality of peer-reviewed published scientific literature of systematic reviews and/or meta-analyses of RCTs pertinent to WP supplementation and T2DM risk factors. This umbrella systematic review followed the PRIOR recommended guide for conducting a comprehensive and rigorous search of the scientific literature of systematic reviews and/or meta-analyses of RCTs pertinent to WP supplementation and T2DM risk factors and reporting the evidence reviewed for this article. In addition, the use of the AMSTAR 2 critical appraisal tool for assessing the methodological quality of included articles is another strength by providing an indication of the confidence, or lack thereof, in the integrity of articles included and reported findings.

Limitations of this review include only including articles published in English language. We are mindful that with the use of WP supplements it is important to report on participant safety and any potential adverse health outcomes. However, among the included articles, only one [33] reported on the effects or associations of WP supplementation on participant safety or adverse health outcomes. In this article, 1 RCT stated 3 and 4 participants in the comparator and WP groups, respectively, reported mild diarrhea, and 2 participants in the WP group reported flatus. No adverse events were reported among the other 4 RCTs included in this article [33]. One other limitation of included articles is that while they reported participant characteristics, they did not specify race or ethnicity.

## Future research recommendations

Based on the available evidence and results from this comprehensive umbrella systematic review of RCTs on the effects of whey protein supplementation on T2DM risk factors, the following suggestions may be considered for future research:

1) Future systematic reviews and/or meta-analyses that include investigating the effects of WP supplementation dose, duration, types, and control or comparator interventions on T2DM risk factors are warranted.

Rationale: Information regarding acute and chronic WP doses, durations, types, or comparator interventions were insufficient to draw conclusions, negating making practical and clinical recommendations. These factors may influence the magnitudes and directionalities of potential responses.

2) Future research on WP supplementation should include reporting on participant safety and any adverse health outcomes.

Rationale: Among the included articles, only 1 of 13 reported on the effects or associations of WP supplementation on participant safety or adverse health outcomes.

3) Future research should include race and ethnicity when reporting participant characteristics.

Rationale: None of the 13 articles included information regarding participant race or ethnicity.

4) Future systematic reviews and/or meta-analyses should utilize a transparent framework, such as GRADE, for providing evidence summaries and rating the certainty of evidence for the effects of WP on T2DM risk factors.

Rationale: Quality of evidence ratings are important when evaluating the certainty of evidence to better inform clinical recommendations and evidence-based practice.

## Conclusions

Among the 13 systematic reviews, including 12 meta-analyses, critically assessed for this umbrella review, no reviews reported any adverse effects of WP on any reported T2DM-related risk factor. Collectively, evidence indicates WP supplementation may improve several clinical indicators of T2DM risk, including multiple measures of glucose control in individuals classified as overweight or with obesity or at risk for or with metabolic syndrome; triglycerides in individuals classified as overweight or with obesity or at risk for or with metabolic syndrome; and blood pressure in individuals classified as overweight or with obesity.

## Author contributions

The authors’ responsibilities were as follows—GC, YW, REB, WWC: designed the research; JBR: conducted the search, GC, YW, REB, EMD, AWB: completed article selection and data collection; GC: wrote the manuscript with editorial assistance from all coauthors; WWC: had primary responsibility for final content; and all authors read and approved the final manuscript.

## Funding

This study was supported by the Whey Protein Research Consortium. The funder had no role in the design, conduction, analysis, or interpretation of this research.

## Data availability

Data described in the manuscript will be made available upon request pending application and approval.

## Conflict of interest

During the time this research was conducted, WWC received funding for research grants, travel or honoraria for scientific presentations, or consulting services from the following organizations: National Institutes of Health, US Department of Agriculture, Beef Checkoff, Foundation for Meat and Poultry Research and Education, Pork Checkoff, North Dakota Beef Commission, Barilla Group, Mushroom Council, National Chicken Council, and the Whey Protein Research Consortium. JBR received grant support from the National Chicken Council and the Whey Protein Research Consortium. REB is currently employed by Archer-Daniels-Midland (ADM); research presented in this article was conducted in a former role and has no connection with ADM. GC, YW, EMD, and AWB report no conflicts of interest.
