# Supplementary material for: Whey Protein Supplementation and Type 2 Diabetes Mellitus Risk Factors: An Umbrella Systematic Review of Randomized Controlled Trials
Source: Curr Dev Nutr. 2023 Oct 14;7(12):102017. doi: 10.1016/j.cdnut.2023.102017 (PMC10709019; doi:10.1016/j.cdnut.2023.102017)
Supplement: Multimedia component 1 [file mmc1.docx]

**Title:** Whey protein supplementation and type 2 diabetes mellitus risk factors: An umbrella systematic review of randomized controlled trials

**First Author:** Gavin Connolly

**Online Supplementary Material**

**Supplementary Table 1:** Database search strategies and number of articles returned

**Supplementary Table 2:** Pre-determined information extracted from the articles that met the inclusion criteria

**Supplemental Table 3.** Randomized controlled trials included among articles

**Supplementary Table 4.** Randomized controlled trials included among articles, and overlap among and between articles that included fasting blood glucose as an outcome

**Supplementary Table 5**. Randomized controlled trials included among articles, and overlap among and between articles that included total cholesterol as an outcome

**Supplementary Table 6.** Randomized controlled trials included among articles, and overlap among and between articles that included low-density lipoprotein cholesterol as an outcome

**Supplementary Table 7.** Randomized controlled trials included among articles, and overlap among and between articles that included triglycerides as an outcome

**Supplementary Table 8.** Randomized controlled trials included among articles, and overlap among and between articles that included body weight as an outcome

**Supplementary Table 9.** Randomized controlled trials included among articles and overlap between articles that included BMI as an outcome

**Supplementary Table 10.** Randomized controlled trials included among articles, and overlap among and between articles that included waist circumference as an outcome

**Supplementary Table 11.** Randomized controlled trials included among articles between articles that included CRP as an outcome

**Supplementary Table 12.** Randomized controlled trials included among articles between articles that included hs-CRP as an outcome

**Supplementary Table 13.1.** AMSTAR2 analysis and ratings for the included articles

**Supplementary Table 13.2.** AMSTAR2 analysis for our umbrella systematic review of systematic reviews and meta-analyses

**Supplementary Table 14.** Subgroup analysis results from included articles

**Supplementary Table 15.** GRADE approach ratings for certainty of evidence included among articles

**Supplemental Table 1**. Database search strategies and number of articles returned.

| **Database** | **Search Terms** | **Filters** | **Number of Articles Returned** |
| --- | --- | --- | --- |
| PubMed | 1. "Whey Proteins"[MeSH Terms:noexp] 2. Protein[Title/Abstract] OR Proteins[Title/Abstract] 3. Whey[Title/Abstract] OR supplement[Title/Abstract] OR supplementation[Title/Abstract] OR supplements[Title/Abstract] 4. #2 AND #3 5. #1 OR #4 6. ((systematic*[tiab] AND (bibliographic*[TIAB] OR literature[tiab] OR review[tiab] OR reviewed[tiab] OR reviews[tiab])) OR (comprehensive*[TIAB] AND (bibliographic*[TIAB] OR literature[tiab])) OR "Evidence report/technology assessment (Summary)"[journal] OR "Evidence report/technology assessment"[journal] OR "cochrane database syst rev"[Journal] OR "integrative literature review"[tiab] OR "integrative research review"[tiab] OR "integrative review"[tiab] OR "research synthesis"[tiab] OR "research integration"[tiab] OR cinahl[tiab] OR embase[tiab] OR medline[tiab] OR psyclit[tiab] OR (psycinfo[tiab] NOT "psycinfo database"[tiab]) OR pubmed[tiab] OR scopus[tiab] OR "web of science"[tiab] OR "data synthesis"[tiab] OR meta-analys*[tiab] OR meta-analyz*[tiab] OR meta-analyt*[tiab] OR metaanalys*[tiab] OR metaanalyz*[tiab] OR metaanalyt*[tiab] OR "meta-analysis as topic"[MeSH:noexp] OR Meta-Analysis[ptyp] OR "Systematic Reviews as Topic"[Mesh] OR "Systematic Review" [Publication Type] OR ((review[tiab] AND (rationale[tiab] OR evidence[tiab])) AND review[pt])) 7. #5 AND #6 8. (("Animals"[MeSH Terms] NOT ("Animals"[MeSH Terms] AND "Humans"[MeSH Terms]))) 9. #7 NOT #8 | English | 1,731 |
| Scopus | 1.( ( TITLE-ABS-KEY ( whey ) ) AND ( TITLE-ABS-KEY ( protein OR proteins ) ) ) OR ( TITLE-ABS-KEY ( protein* W/4 supplement* ) )    2.( TITLE-ABS-KEY ( systematic* W/3 review* ) ) OR ( TITLE-ABS-KEY ( systematic* W/3 bibliographic* ) ) OR ( TITLE-ABS-KEY ( systematic* W/3 literature ) ) OR ( TITLE-ABS-KEY ( comprehensive* W/3 literature ) ) OR ( TITLE-ABS-KEY ( comprehensive* W/3 bibliographic* ) ) OR ( TITLE-ABS-KEY ( integrative W/3 review ) ) OR ( SRCTITLE ( {Cochrane Database of Systematic Reviews} ) ) OR ( TITLE-ABS-KEY ( information W/2 synthesis ) ) OR ( TITLE-ABS-KEY ( data W/2 synthesis ) ) OR ( TITLE-ABS-KEY ( data W/2 extract* ) ) OR ( TITLE-ABS-KEY ( medline OR pubmed OR psyclit OR cinahl OR ( psycinfo AND NOT {psycinfo database} ) OR {web of science} OR scopus OR embase ) ) OR ( TITLE-ABS-KEY ( meta-analy* OR metaanaly* ) )  3.#1 AND #2 | English | 674 |
| Cochrane Library  (reviews) | 1. MeSH descriptor: [Whey Proteins] this term only 2. (protein OR proteins):ti,ab,kw 3. (supplement or supplements or supplementation or whey):ti,ab,kw 4. #2 AND #3   #1 OR #4 | N/A | 80 |
| Cochrane Library  (for trials) | 1. (systematic*):ti,ab,kw 2. (bibliographic* OR literature OR review OR reviewed OR reviews):ti,ab,kw 3. #1 AND #2 4. (comprehensive*):ti,ab,kw 5. (bibliographic* OR literature):ti,ab,kw 6. #4 AND #5 7. ("integrative literature review" OR "integrative research review" OR "integrative review" OR “research synthesis” OR “research integration” OR cinahl OR embase OR medline OR psyclit OR pubmed OR scopus OR “web of science” OR “data synthesis” OR meta-analys* OR meta-analyz* OR meta-analyt* OR metaanalys* OR metaanalyz*OR metaanalyt*):ti,ab,kw 8. (psycinfo NOT “psycinfo database”):ti,ab,kw 9. MeSH descriptor: [Meta-Analysis as Topic] this term only 10. (Meta-Analysis):pt 11. #7 OR #8 OR #9 OR #10 12. (review):ti,ab,kw 13. rationale OR evidence):ti,ab,kw 14. #12 AND #13 15. (review):pt 16. #14 AND #15 17. #3 OR #6 OR #11 OR #16 18. MeSH descriptor: [Whey Proteins] this term only 19. (protein OR proteins):ti,ab,kw 20. (supplement or supplements or supplementation or whey):ti,ab,kw 21. #19 AND #20 22. #18 OR #21   #17 AND #22 | N/A | 176 |
| CINAHL | 1. (MH "Whey Proteins") 2. (TI whey OR AB whey OR MW whey ) 3. TI ( protein OR proteins ) OR AB ( protein OR proteins ) OR MW ( protein OR proteins ) 4. S2 AND S3 5. (TI (protein* n4 supplement*) OR AB (protein* n4 supplement*) OR MW (protein* n4 supplement*) 6. S1 OR S4 OR S5 7. (TI (systematic* n3 review*)) or (AB (systematic* n3 review*)) or (TI (systematic* n3 bibliographic*)) or (AB (systematic* n3 bibliographic*)) or (TI (systematic* n3 literature)) or (AB (systematic* n3 literature)) or (TI (comprehensive* n3 literature)) or (AB (comprehensive* n3 literature)) or (TI (comprehensive* n3 bibliographic*)) or (AB (comprehensive* n3 bibliographic*)) or (TI (integrative n3 review)) or (AB (integrative n3 review)) or (JN “Cochrane Database of Systematic Reviews”) or (TI (information n2 synthesis)) or (TI (data n2 synthesis)) or (AB (information n2 synthesis)) or (AB (data n2 synthesis)) or (TI (data n2 extract*)) or (AB (data n2 extract*)) or (TI (medline or pubmed or psyclit or cinahl or (psycinfo not “psycinfo database”) or “web of science” or scopus or embase)) or (AB (medline or pubmed or psyclit or cinahl or (psycinfo not “psycinfo database”) or “web of science” or scopus or embase)) or (MH “Systematic Review”) or (MH “Meta Analysis”) or (TI (meta-analy* or metaanaly*)) or (AB (meta-analy* or metaanaly*)) 8. S6 AND S7 | English,  Academic Journals | 248 |
| SPORTDiscus | 1. TI whey OR AB whey OR KW whey 2. TI ( protein or protein ) OR AB ( Protein OR proteins ) OR KW ( Protein OR proteins ) 3. S1 AND S2 4. TI (protein* n4 supplement*) OR AB (protein* n4 supplement*) OR KW (protein* n4 supplement*) 5. S3 OR S4 6. (TI (systematic* n3 review*)) or (AB (systematic* n3 review*)) or (TI (systematic* n3 bibliographic*)) or (AB (systematic* n3 bibliographic*)) or (TI (systematic* n3 literature)) or (AB (systematic* n3 literature)) or (TI (comprehensive* n3 literature)) or (AB (comprehensive* n3 literature)) or (TI (comprehensive* n3 bibliographic*)) or (AB (comprehensive* n3 bibliographic*)) or (TI (integrative n3 review)) or (AB (integrative n3 review)) or (JN “Cochrane Database of Systematic Reviews”) or (TI (information n2 synthesis)) or (TI (data n2 synthesis)) or (AB (information n2 synthesis)) or (AB (data n2 synthesis)) or (TI (data n2 extract*)) or (AB (data n2 extract*)) or (TI (medline or pubmed or psyclit or cinahl or (psycinfo not “psycinfo database”) or “web of science” or scopus or embase)) or (AB (medline or pubmed or psyclit or cinahl or (psycinfo not “psycinfo database”) or “web of science” or scopus or embase)) or (TI (meta-analy* or metaanaly*)) or (AB (meta-analy* or metaanaly*)) 7. S5 AND S6 | English, Journal Articles | 55 |

**Supplemental Table 2:** Pre-determined information extracted from the articles that met the inclusion criteria. Data were extracted in Covidence.

| **Data Extracted** |
| --- |
| **General information** |
| Study ID (DOI and/or PMID) |
| Title |
| Geographical location of research |
| Notes |
| **Methods** |
| Aim of study |
| Primary outcome |
| Secondary outcome |
| Start date of search |
| End date of search |
| Study funding sources |
| Possible conflicts of interest for study authors |
| Number of databases search |
| Number of studies added manually (not through primary search) |
| Language requirement? |
| Study quality assessment? |
| Study quality results |
| **Participant and intervention information** |
| Population description |
| Inclusion criteria |
| Exclusion criteria |
| Total number of RCTs included |
| Total number of participants |
| Number of RCTs investigating whey protein |
| Type(s) of whey protein used (Ex. Isolate) |
| Dosage of whey (in grams) – For example, if a review included a total of 20 studies, the dosage could be as follows: 3 studies used 20 grams, 4 studies used 25 grams, and 4 that used 30 grams |
| Dosage range of whey in studies that are included in review (range: lowest-highest) |
| Number of participants in studies investigating whey protein  Duration of included studies in weeks (range: shortest to longest) |
| Sex |
| Age range of participants |
| Mean age of participants (if provided) |
| Health status |
| BMI status |
| Exercise |
| Energy restriction |
| **Results** |
| ***T2DM Risk Factors*** |
| Fasting glucose WMD (95% CI) |
| Fasting insulin WMD (95% CI) |
| Postprandial glucose WMD (95% CI) |
| 24–h CGM iAUC WMD (95% CI) |
| HbA1c WMD (95% CI) |
| Total cholesterol WMD (95% CI) |
| LDL–C WMD (95% CI) |
| HDL–C WMD (95% CI) |
| Triglycerides WMD (95% CI) |
| CRP WMD (95% CI) |
| hs-CRP WMD (95% CI) |
| Systolic blood pressure WMD (95% CI) |
| Diastolic blood pressure WMD (95% CI) |
| Body weight WMD (95% CI) |
| BMI WMD (95% CI)  Waist circumference WMD (95% CI) |

BMI, body mass index; CGM, continuous glucose monitor; CI, confidence interval; DOI; digital object identifier; HbA1c, hemoglobin A1c; hs-CRP, high-senstitivity C-reactive protein; HDL-C, high-density lipoprotein cholesterol; iAUC, incremental area under the curve; LDL-C, low-density lipoprotein cholesterol; PMID, PubMed Identifier; RCT, randomized controlled trial; T2DM, type 2 diabetes mellitus; WMD, weighted mean difference.

**Supplemental Table 3.** Randomized controlled trials included among articles in this umbrella systamtic review.

| **RCTs included among articles** | **Amirani et al. (2020) (1)** | **Badely et al. (2019)**  **(2)** | **Bergia et al. (2018)**  **(3)** | **Blair et al. (2020)**  **(4)** | **Chiang et al. (2022)**  **(5)** | **Kuo et al. (2022)**  **(6)** | **Miller et al. (2014)**  **(7)** | **Piri Damaghi et al. (2022)**  **(8)** | **Prokopidis et al. (2022)**  **(9)** | **Sepandi et al. (2022)**  **(10)** | **Wirunsawanya et al.**  **(2018)**  **(11)** | **Zhang et al. (2016)**  **(12)** | **Zhou et al. (2015)**  **(13)** |
| --- | --- | --- | --- | --- | --- | --- | --- | --- | --- | --- | --- | --- | --- |
| Arciero et al. (2016) (14) |  | ✓ |  |  |  |  |  |  |  |  |  |  |  |
| Adechian et al. (2012) (15) |  |  | ✓ |  |  |  |  |  |  |  |  |  |  |
| Aldrich et al. (2011) (16) |  | ✓ |  |  |  |  |  |  |  |  |  |  |  |
| Baer et al. (2011) (17) |  |  |  |  |  |  | ✓ | ✓ |  |  |  |  |  |
| Beavers et al. (2015) (18) |  | ✓ |  |  |  |  |  |  |  |  |  |  |  |
| Bell et al. (2017) (19) |  | ✓ |  |  |  |  |  |  |  | ✓ |  |  |  |
| Berthold et al. (2011) (20) |  | ✓ |  |  |  |  |  |  |  |  |  | ✓ |  |
| Bo et al. (2019) (21) |  |  |  |  |  |  |  |  | ✓ |  |  |  |  |
| Bohl et al. (2015) (22) |  |  |  |  |  |  |  |  | ✓ |  |  |  |  |
| Bohl et al. (2016) (23) |  | ✓ |  |  |  |  |  |  |  |  |  |  |  |
| Brown et al. (2004) (24) |  |  |  |  |  |  |  | ✓ |  |  |  |  |  |
| Brown et al. (2020) (25) |  |  |  |  |  |  |  |  |  | ✓ |  |  |  |
| Bumrungpert et al. (2018) (26) |  |  |  |  |  |  |  |  | ✓ |  |  |  |  |
| Burke et al. (2001) (27) |  |  |  |  |  |  | ✓ |  |  |  |  |  |  |
| Candow et al. (2006) (28) |  |  |  |  |  |  |  | ✓ |  |  |  |  |  |
| Chalé et al. (2013) (29) |  |  |  |  |  |  |  |  |  | ✓ |  |  |  |
| Chiu et al. (2014) (30) |  |  |  |  |  |  |  |  |  |  |  | ✓ |  |
| Claessens et al. (2009) (31) | ✓ |  |  |  |  |  | ✓ |  |  | ✓ |  | ✓ |  |
| Cribb et al. (2006) (32) |  |  |  |  |  |  | ✓ |  |  |  |  |  |  |
| Cribb et al. (2007) (33) |  |  |  |  |  |  | ✓ |  |  |  |  |  |  |
| Demling et al. (2000) (34) |  |  |  |  |  |  | ✓ |  |  | ✓ |  |  |  |
| Denysschen et al. (2009) (35) | ✓ | ✓ |  |  |  |  | ✓ | ✓ |  | ✓ |  | ✓ |  |
| Derosa et al. (2020) (36) |  |  |  |  |  |  |  |  | ✓ |  |  |  |  |
| Duff et al. (2014) (37) |  |  | ✓ |  |  |  |  |  | ✓ |  |  |  | ✓ |
| Eliot et al. (2008) (38) |  |  |  |  |  |  | ✓ |  |  | ✓ |  |  |  |
| Fekete et al. (2016) (39) | ✓ | ✓ |  |  |  |  |  |  | ✓ |  |  |  |  |
| Fernandes et al. (2018) (40) |  | ✓ |  |  |  |  |  |  | ✓ | ✓ |  |  |  |
| Figueroa et al. (2013) (41) |  | ✓ |  |  |  |  |  |  |  |  | ✓ |  |  |
| Fluegel et al. (2010) (42) |  | ✓ |  |  |  |  |  |  |  |  |  |  |  |
| Frestedt et al. (2008) (43) | ✓ |  |  |  |  |  |  |  |  |  | ✓ | ✓ |  |
| Gaffney et al. (2018) (44) | ✓ |  |  |  |  |  |  |  |  |  |  |  |  |
| Gordon et al. (2008) (45) |  |  | ✓ |  |  |  |  |  |  |  |  |  |  |
| Gouni-Berthold et al. (2012) (46) |  | ✓ |  |  |  |  |  |  |  |  |  | ✓ | ✓ |
| Gryson et al. (2014) (47) |  | ✓ |  |  |  |  |  |  |  |  |  |  |  |
| Gulati et al. (2017) (48) |  | ✓ |  |  |  |  |  |  |  |  |  |  |  |
| Haidari et al. (2019) (49) |  |  |  |  |  |  |  |  |  | ✓ |  |  |  |
| Hambre et al. (2012) (50) |  |  |  |  |  |  |  |  |  | ✓ | ✓ | ✓ |  |
| Hassanzadeh-Rostami et al. (2020) (51) |  |  |  |  |  |  |  |  |  | ✓ |  |  |  |
| Hector et al. (2015) (52) |  |  |  |  |  |  |  | ✓ |  |  | ✓ |  |  |
| Herda et al. (2013) (53) |  |  |  |  |  |  |  |  |  | ✓ |  |  |  |
| Herda et al. (2021) (54) |  |  |  |  |  | ✓ |  |  |  |  |  |  |  |
| Hodgson et al. (2011) (55) |  | ✓ |  |  |  | ✓ |  |  |  |  |  |  |  |
| Holm et al. (2008) (56) |  |  | ✓ |  |  |  |  |  |  |  |  |  |  |
| Holwerda et al. (2018) (57) |  |  |  |  |  |  |  |  |  | ✓ |  |  |  |
| Jakubowicz et al. (2017) (58) | ✓ |  |  |  | ✓ |  |  |  |  |  |  |  |  |
| Kasim-Karakis et al. (2009) (59) | ✓ |  |  |  |  |  |  |  |  | ✓ |  |  |  |
| Kemmler et al. (2015) (60) |  | ✓ |  |  |  |  |  |  |  |  |  |  |  |
| Kemmler et al. (2018) (61) | ✓ | ✓ |  |  |  |  |  |  |  |  |  |  |  |
| Keogh and Clifton (2008) (62) |  | ✓ | ✓ |  |  |  | ✓ |  |  | ✓ |  | ✓ |  |
| Kerstetter et al. (2015) (63) |  |  |  |  |  |  |  |  |  | ✓ |  |  |  |
| King et al. (2018) (64) |  |  |  |  | ✓ |  |  |  |  |  |  |  |  |
| Kinsey et al. (2014) (65) |  | ✓ |  |  |  |  |  |  |  |  | ✓ |  |  |
| Kirk et al. (2019) (66) |  |  |  |  |  |  |  |  |  | ✓ |  |  |  |
| Kirk et al. (2021) (67) |  |  |  |  |  |  |  |  | ✓ |  |  |  |  |
| Kjølbæk et al. (2017) (68) | ✓ | ✓ | ✓ |  |  |  |  | ✓ |  |  |  |  |  |
| Larsen et al. (2018) (69) | ✓ | ✓ |  |  |  |  |  |  |  |  |  |  |  |
| Laviolette et al. (2010) (70) |  |  |  |  |  |  |  |  | ✓ |  |  |  | ✓ |
| Lee et al. (2007) (71) | ✓ | ✓ |  |  |  |  |  |  |  |  |  | ✓ | ✓ |
| Li et al. (2021) (72) |  |  |  |  |  |  |  |  |  | ✓ |  |  |  |
| Lopes Gomes et al. (2017) (73) | ✓ |  |  |  |  |  |  |  |  |  |  |  |  |
| Lynch et al. (2019) (74) |  |  |  |  |  |  |  |  |  | ✓ |  |  |  |
| Ma et al. (2015) (75) |  |  |  |  | ✓ |  |  |  |  |  |  |  |  |
| Martens et al. (2015) (76) |  |  | ✓ |  |  |  |  |  |  |  |  |  |  |
| Matsuoka et al. (2017) (77) |  | ✓ |  |  |  |  |  |  |  |  |  |  |  |
| McAdam et al. (2018) (78) |  |  |  |  |  |  |  |  |  | ✓ |  |  |  |
| Mobley et al. (2017) (79) |  |  |  |  |  |  |  | ✓ |  |  |  |  |  |
| Moeller et al. (2003) (80) |  |  |  |  |  |  | ✓ |  |  |  |  |  |  |
| Mohammadi-Sartang et al. (2018) (81) | ✓ | ✓ |  |  |  |  |  |  |  |  |  |  |  |
| Mojtahedi et al. (2011) (82) |  |  | ✓ |  |  |  | ✓ |  |  | ✓ |  |  |  |
| Mori and Takudo (2018) (83) |  |  |  |  |  | ✓ |  |  |  | ✓ |  |  |  |
| Nabuco et al. (2019)a (84) |  |  |  |  |  |  |  |  | ✓ | ✓ |  |  |  |
| Nabuco et al. (2019)b (85) |  | ✓ |  |  |  |  |  |  |  | ✓ |  |  |  |
| Ormsbee et al. (2015) (86) | ✓ | ✓ |  |  |  |  |  |  |  | ✓ |  |  |  |
| Padhi et al. (2015) (87) |  | ✓ |  |  |  |  |  |  |  |  |  |  |  |
| Pal et al. (2010)a (88) | ✓ | ✓ |  |  |  |  | ✓ |  |  |  | ✓ | ✓ |  |
| Pal et al. (2010)b (89) |  |  |  |  |  |  |  |  |  |  | ✓ |  | ✓ |
| Pal et al. (2010)c (90) |  |  |  |  |  |  |  |  |  |  | ✓ |  |  |
| Pal et al. (2014) (91) |  |  |  |  |  |  |  |  |  | ✓ |  |  |  |
| Petyaev et al. (2012) (92) | ✓ | ✓ |  |  |  |  |  |  |  |  |  | ✓ | ✓ |
| Piccolo et al. (2015) (93) |  | ✓ |  |  |  |  |  |  |  | ✓ | ✓ |  |  |
| Pins et al. (2006) (94) |  |  |  |  |  |  |  |  |  |  |  |  | ✓ |
| Rakvaag et al. (2019) (95) | ✓ |  |  |  |  |  |  |  | ✓ |  |  |  |  |
| Reidy et al. (2016) (96) |  |  |  |  |  |  |  | ✓ |  |  |  |  |  |
| Reidy et al. (2017) (97) |  |  |  |  |  |  |  | ✓ |  |  |  |  |  |
| Reimer et al. (2017) (98) |  | ✓ |  |  |  |  |  |  |  |  |  |  |  |
| Roberson et al. (2021) (99) |  |  |  |  |  |  |  |  |  | ✓ |  |  |  |
| Rondanelli et al. (2016) (100) |  |  |  |  |  |  |  |  | ✓ |  |  |  |  |
| Sahathevan et al. (2018) (101) |  |  |  |  |  |  |  |  |  | ✓ |  |  |  |
| Sattler et al. (2008) (102) |  |  |  |  |  |  |  |  |  | ✓ |  |  |  |
| Sohrabi et al. (2016) (103) |  |  |  |  |  |  |  |  | ✓ |  |  |  |  |
| Stragier et al. (2016) (104) |  |  | ✓ |  |  |  |  |  |  |  |  |  |  |
| Stojkovic et al. (2017) (105) |  |  |  |  |  |  |  |  | ✓ | ✓ |  |  |  |
| Sugawara et al. (2012) (106) |  |  |  |  |  |  |  |  |  |  |  |  | ✓ |
| Sukumar et al. (2011) (107) |  |  | ✓ |  |  |  |  |  |  |  |  |  |  |
| Tahavorgar et al. (2015) (108) |  | ✓ |  |  |  |  |  |  |  | ✓ |  |  |  |
| Taylor et al. (2016) (109) |  |  | ✓ |  |  |  |  |  |  |  |  |  |  |
| Tovar et al. (2012)(110) | ✓ |  |  |  |  |  |  |  |  |  |  |  |  |
| Tovar et al. (2016) (111) | ✓ | ✓ |  |  |  |  |  |  |  |  |  |  |  |
| Vatani and Golzar (2012) (112) | ✓ | ✓ |  |  |  |  |  |  |  | ✓ |  | ✓ |  |
| Verreijen et al. (2015) (113) |  |  | ✓ |  |  |  |  |  |  | ✓ | ✓ |  |  |
| Verreijen et al. (2017) (114) |  | ✓ |  |  |  |  |  |  |  |  |  |  |  |
| Volek et al. (2013) (115) |  |  |  |  |  |  |  | ✓ |  | ✓ |  |  |  |
| Watson et al. (2019) (116) | ✓ |  |  |  | ✓ |  |  |  |  |  |  |  |  |
| Weinheimer et al. (2012) (117) |  |  | ✓ |  |  |  | ✓ |  | ✓ | ✓ |  | ✓ | ✓ |
| Weisgarber et al. (2012) (118) |  |  |  |  |  |  | ✓ |  |  |  |  |  |  |
| Wu et al. (2016) (119) |  |  |  |  | ✓ |  |  |  |  |  |  |  |  |
| Yang et al. (2019) (120) | ✓ |  |  |  |  |  |  |  |  |  |  |  |  |
| Zhu et al. (2015) (121) |  |  |  |  |  | ✓ |  |  |  |  |  |  |  |

RCTs, randomized controlled trials; ✓, included within review article.

**Supplementary Table 4.** Randomized controlled trials included among articles, and overlap among and between articles that included fasting blood glucose as an outcome.

| **RCTs included among articles** | **Amirani et al. (2020)**  **(1)** | **Badely et al. (2019)**  **(2)** | **Wirunsawanya et al.**  **(2018)**  **(11)** | **Overlap among all articles** | **Overlap between Amirani et al. (1) & Badely et al. (2)** | **Overlap between Amirani et al. (1) & Wirunsawanya et al. (11)** | **Overlap between Badely et al. (2) & Wirunsawanya et al. (11)** |
| --- | --- | --- | --- | --- | --- | --- | --- |
| Total number of RCTs | 17 | 16 | 4 | 1 | 1 | 1 | 3 |
| Arciero et al. (2016) (14) |  | ✓ |  |  |  |  |  |
| Beavers et al. (2015) (18) |  | ✓ |  |  |  |  |  |
| Bohl et al. (2016) (22) |  | ✓ |  |  |  |  |  |
| Claessens et al. (2009) (31) | ✓ |  |  |  |  |  |  |
| Fekete et al. (2016) (39) | ✓ |  |  |  |  |  |  |
| Fluegel et al. (2010) (42) |  | ✓ |  |  |  |  |  |
| Gaffney et al. (2018) (44) | ✓ |  |  |  |  |  |  |
| Gouni-Berthold et al. (2012) (46) |  | ✓ |  |  |  |  |  |
| Gulati et al. (2017) (48) |  | ✓ |  |  |  |  |  |
| Jakubowicz et al. (2017) (58) | ✓ |  |  |  |  |  |  |
| Kasim-Karakis et al. (2009) (59) | ✓ |  |  |  |  |  |  |
| Kemmler et al. (2015) (60) |  | ✓ |  |  |  |  |  |
| Keogh and Clifton (2008) (62) |  | ✓ |  |  |  |  |  |
| Kinsey et al. (2014) (65) |  | ✓ | ✓ |  |  |  | x |
| Kjølbæk et al. (2017) (68) | ✓ |  |  |  |  |  |  |
| Larsen et al. (2018) (69) | ✓ |  |  |  |  |  |  |
| Lee et al. (2007) (71) | ✓ | ✓ |  |  | x |  |  |
| Lopes Gomes et al. (2017) (73) | ✓ |  |  |  |  |  |  |
| Mohammadi-Sartang et al. (2018) (81) | ✓ | ✓ |  |  |  |  |  |
| Ormsbee et al. (2015) (86) | ✓ |  |  |  |  |  |  |
| Pal et al. (2010)a (88) | ✓ | ✓ | ✓ | x | x | x | x |
| Pal et al. (2010)b (89) |  |  | ✓ |  |  |  |  |
| Pal et al. (2010)c (90) |  |  | ✓ |  |  |  |  |
| Piccolo et al. (2015) (93) |  | ✓ | ✓ |  |  | x |  |
| Rakvaag et al. (2019) (95) | ✓ |  |  |  |  |  |  |
| Tahavorgar et al. (2015) (108) |  | ✓ |  |  |  |  |  |
| Tovar et al. (2012) (110) | ✓ |  |  |  |  |  |  |
| Tovar et al. (2016) (111) | ✓ | ✓ |  |  | x |  |  |
| Vatani and Golzar (2012) (112) | ✓ | ✓ |  |  | x |  |  |
| Yang et al. (2019) (120) | ✓ |  |  |  |  |  |  |

RCTs, randomized controlled trials; ✓, included within review article; x, overlap among or between articles.

**Supplementary Table 5**. Randomized controlled trials included among articles, and overlap among and between articles that included total cholesterol as an outcome.

| **RCTs included among articles** | **Amirani et al. (2020)**  **(1)** | **Wirunsawanya et al.**  **(2018)**  **(11)** | **Zhang et al. (2016)**  **(12)** | **Overlap among all articles** | **Overlap between Amirani et al. (1) & Wirunsawanya et al. (11)** | **Overlap between Amirani et al. (1) & Zhang et al. (12)** | **Overlap between Wirunsawanya et al. (11) & Zhang et al. (12)** |
| --- | --- | --- | --- | --- | --- | --- | --- |
| Total number of RCTs | 18 | 6 | 13 | 2 | 2 | 7 | 3 |
| Berthold et al. (2011) (20) |  |  | ✓ |  |  |  |  |
| Chiu et al. (2014) (30) |  |  | ✓ |  |  |  |  |
| Claessens et al. (2009) (31) | ✓ |  | ✓ |  |  | x |  |
| Denysschen et al. (2009) (35) | ✓ |  | ✓ |  |  | x |  |
| Fekete et al. (2016) (39) | ✓ |  |  |  |  |  |  |
| Frestedt et al. (2008) (43) | ✓ | ✓ | ✓ | x | x | x | x |
| Gouni-Berthold et al. (2012) (46) |  |  | ✓ |  |  |  |  |
| Hambre et al. (2012) (50) |  | ✓ | ✓ |  |  |  | x |
| Kasim-Karakis et al. (2009) (59) | ✓ |  |  |  |  |  |  |
| Keogh and Clifton (2008) (62) |  |  | ✓ |  |  |  |  |
| Kinsey et al. (2014) (65) |  | ✓ |  |  |  |  |  |
| Kjølbæk et al. (2017) (68) | ✓ |  |  |  |  |  |  |
| Larsen et al. (2018) (69) | ✓ |  |  |  |  |  |  |
| Lee et al. (2007) (71) | ✓ |  | ✓ |  |  | x |  |
| Lopes Gomes et al. (2017) (73) | ✓ |  |  |  |  |  |  |
| Mohammadi-Sartang et al. (2018) (81) | ✓ |  |  |  |  |  |  |
| Ormsbee et al. (2015) (86) | ✓ |  |  |  |  |  |  |
| Pal et al. (2010)a (88) | ✓ | ✓ | ✓ | x | x | x | x |
| Pal et al. (2010)b (89) |  | ✓ |  |  |  |  |  |
| Petyaev et al. (2012) (92) | ✓ |  | ✓ |  |  | x |  |
| Piccolo et al. (2015) (93) |  | ✓ |  |  |  |  |  |
| Rakvaag et al. (2019) (95) | ✓ |  |  |  |  |  |  |
| Tovar et al. (2012) (110) | ✓ |  |  |  |  |  |  |
| Tovar et al. (2016) (111) | ✓ |  |  |  |  |  |  |
| Vatani and Golzar (2012) (112) | ✓ |  | ✓ |  |  | x |  |
| Weinheimer et al. (2012) (117) |  |  | ✓ |  |  |  |  |
| Yang et al. (2019) (120) | ✓ |  |  |  |  |  |  |

RCTs, randomized controlled trials; ✓, included within review article; x, overlap among or between articles.

**Supplementary Table 6.** Randomized controlled trials included among articles, and overlap among and between articles that included low-density lipoprotein cholesterol as an outcome.

| **RCTs included among articles** | **Amirani et al. (2020)**  **(1)** | **Wirunsawanya et al.**  **(2018)**  **(11)** | **Zhang et al. (2016)**  **(12)** | **Overlap among all articles** | **Overlap between Amirani et al. (1) & Wirunsawanya et al. (11)** | **Overlap between Amirani et al. (1) & Zhang et al. (12)** | **Overlap between Wirunsawanya et al. (11) & Zhang et al. (12)** |
| --- | --- | --- | --- | --- | --- | --- | --- |
| Total number of RCTs | 15 | 6 | 12 | 2 | 2 | 6 | 3 |
| Berthold et al. (2011) (20) |  |  | ✓ |  |  |  |  |
| Chiu et al. (2014) (30) |  |  | ✓ |  |  |  |  |
| Claessens et al. (2009) (31) | ✓ |  | ✓ |  |  | x |  |
| Fekete et al. (2016) (39) | ✓ |  |  |  |  |  |  |
| Frestedt et al. (2008) (43) | ✓ | ✓ | ✓ | x | x | x | x |
| Gouni-Berthold et al. (2012) (46) |  |  | ✓ |  |  |  |  |
| Hambre et al. (2012) (50) |  | ✓ | ✓ |  |  |  | x |
| Keogh and Clifton (2008) (62) |  |  | ✓ |  |  |  |  |
| Kinsey et al. (2014) (65) |  | ✓ |  |  |  |  |  |
| Kjølbæk et al. (2017) (68) | ✓ |  |  |  |  |  |  |
| Lee et al. (2007) (71) | ✓ |  | ✓ |  |  | x |  |
| Lopes Gomes et al. (2017) (73) | ✓ |  |  |  |  |  |  |
| Mohammadi-Sartang et al. (2018) (81) | ✓ |  |  |  |  |  |  |
| Ormsbee et al. (2015) (86) | ✓ |  |  |  |  |  |  |
| Pal et al. (2010)a (88) | ✓ | ✓ | ✓ | x | x | x | x |
| Pal et al. (2010)b (89) |  | ✓ |  |  |  |  |  |
| Petyaev et al. (2012) (92) | ✓ |  | ✓ |  |  | x |  |
| Piccolo et al. (2015) (93) |  | ✓ |  |  |  |  |  |
| Rakvaag et al. (2019) (95) | ✓ |  |  |  |  |  |  |
| Tovar et al. (2012) (110) | ✓ |  |  |  |  |  |  |
| Tovar et al. (2016) (111) | ✓ |  |  |  |  |  |  |
| Vatani and Golzar (2012) (112) | ✓ |  | ✓ |  |  | x |  |
| Weinheimer et al. (2012) (117) |  |  | ✓ |  |  |  |  |
| Yang et al. (2019) (120) | ✓ |  |  |  |  |  |  |

RCTs, randomized controlled trials; ✓, included within review article; x, overlap among or between articles.

**Supplementary Table 7.** Randomized controlled trials included among articles, and overlap among and between articles that included triglycerides as an outcome.

| **RCTs included among articles** | **Amirani et al. (2020)**  **(1)** | **Badely et al. (2019)**  **(2)** | **Wirunsa-wanya et al.**  **(2018)**  **(11)** | **Zhang et al. (2016)**  **(12)** | **Overlap among all articles** | **Overlap between Amirani et al. (1) & Badely et al. (2)** | **Overlap between Amirani et al. (1) & Wirunsa-wanya et al. (11)** | **Overlap between Amirani et al. (1) & Zhang et al. (12)** | **Overlap between Badely et al. (2) & Wirunsa-wanywa et al. (11)** | **Overlap between Badely et al. (2) & Zhang et al. (12)** | **Overlap between Wirunsawanya et al. (11) & Zhang et al. (12)** |
| --- | --- | --- | --- | --- | --- | --- | --- | --- | --- | --- | --- |
| Total number of RCTs | 18 | 27 | 6 | 13 | 1 | 10 | 2 | 7 | 2 | 9 | 5 |
| Arciero et al. (2016) (14) |  | ✓ |  |  |  |  |  |  |  |  |  |
| Beavers et al. (2015) (18) |  | ✓ |  |  |  |  |  |  |  |  |  |
| Bell et al. (2017) (19) |  | ✓ |  |  |  |  |  |  |  |  |  |
| Berthold et al. (2011) (20) |  | ✓ |  | ✓ |  |  |  |  |  | x |  |
| Chiu et al. (2014) (30) |  |  |  | ✓ |  |  |  |  |  |  |  |
| Claessens et al. (2009) (31) | ✓ |  |  | ✓ |  |  |  | x |  |  |  |
| Denysschen et al. (2009) (35) | ✓ | ✓ |  | ✓ |  | x |  | x |  | x |  |
| Fekete et al. (2016) (39) | ✓ | ✓ |  |  |  | x |  |  |  |  |  |
| Fernandes et al. (2018) (40) |  | ✓ |  |  |  |  |  |  |  |  |  |
| Fluegel et al. (2010) (42) |  | ✓ |  |  |  |  |  |  |  |  |  |
| Frestedt et al. (2008) (43) | ✓ |  | ✓ | ✓ |  |  | x | x |  |  | x |
| Gouni-Berthold et al. (2012) (46) |  | ✓ |  | ✓ |  |  |  |  |  | x |  |
| Gryson et al. (2014) (47) |  | ✓ |  |  |  |  |  |  |  |  |  |
| Gulati et al. (2017) (48) |  | ✓ |  |  |  |  |  |  |  |  |  |
| Hambre et al. (2012) (50) |  |  | ✓ | ✓ |  |  |  |  |  |  | x |
| Kasim-Karakis et al. (2009) (59) | ✓ |  |  |  |  |  |  |  |  |  |  |
| Kemmler et al. (2015) (60) |  | ✓ |  |  |  |  |  |  |  |  |  |
| Kemmler et al. (2018) (61) | ✓ | ✓ |  |  |  | x |  |  |  |  |  |
| Keogh and Clifton (2008) (62) |  | ✓ |  | ✓ |  |  |  |  |  | x |  |
| Kinsey et al. (2014) (65) |  | ✓ | ✓ |  |  |  |  |  | x |  |  |
| Kjølbæk et al. (2017) (68) | ✓ | ✓ |  |  |  | x |  |  |  |  |  |
| Lee et al. (2007) (71) | ✓ | ✓ |  | ✓ |  | x |  | x |  | x |  |
| Lopes Gomes et al. (2017) (73) | ✓ |  |  |  |  |  |  |  |  |  |  |
| Matsuoka et al. (2017) (77) |  | ✓ |  |  |  |  |  |  |  |  |  |
| Mohammadi-Sartang et al. (2018) (81) | ✓ | ✓ |  |  |  | x |  |  |  |  |  |
| Ormsbee et al. (2015) (86) | ✓ |  |  |  |  |  |  |  |  |  |  |
| Padhi et al. (2015) (87) |  | ✓ |  |  |  |  |  |  |  |  |  |
| Pal et al. (2010)a (88) | ✓ | ✓ | ✓ | ✓ | x | x | x | x | x | x | x |
| Pal et al. (2010)b (89) |  |  | ✓ |  |  |  |  |  |  |  |  |
| Petyaev et al. (2012) (92) | ✓ | ✓ |  | ✓ |  | x |  | x |  | x |  |
| Piccolo et al. (2015) (93) |  | ✓ | ✓ |  |  |  |  |  |  | x |  |
| Pins et al. (2006) (94) |  | ✓ |  |  |  |  |  |  |  |  |  |
| Rakvaag et al. (2019) (95) | ✓ |  |  |  |  |  |  |  |  |  |  |
| Tahavorgar et al. (2015) (108) |  | ✓ |  |  |  |  |  |  |  |  |  |
| Tovar et al. (2012) (110) | ✓ |  |  |  |  |  |  |  |  |  |  |
| Tovar et al. (2016) (111) | ✓ | ✓ |  |  |  | x |  |  |  |  |  |
| Vatani and Golzar (2012) (112) | ✓ | ✓ |  | ✓ |  | x |  | x |  | x |  |
| Weinheimer et al. (2012) (117) |  |  |  | ✓ |  |  |  |  |  |  |  |
| Yang et al. (2019) (120) | ✓ |  |  |  |  |  |  |  |  |  |  |

RCTs, randomized controlled trials; ✓, included within review article; x, overlap among or between articles.

**Supplementary Table 8.** Randomized controlled trials included among articles, and overlap

among and between articles that included body weight as an outcome.

| **RCTs included among articles** | **Bergia et al. (2018)**  **(3)** | **Kuo et al. (2022)**  **(6)** | **Miller et al. (2014)**  **(7)** | **Piri Damaghi et al.**  **(2022)**  **(8)** | **Sepandi et al. (2022)**  **(10)** | **Wirunsawanya et al.**  **(2018)**  **(11)** |  |
| --- | --- | --- | --- | --- | --- | --- | --- |
| Total number of RCTs | 12 | 4 | 13 | 6 | 35 | 7 |  |
| Adechian et al. (2012) (15) | ✓ |  |  |  |  |  |  |
| Baer et al. (2011) (17) |  |  | ✓ | ✓ |  |  |  |
| Bell et al. (2017) (19) |  |  |  |  | ✓ |  |  |
| Brown et al. (2020) (25) |  |  |  |  | ✓ |  |  |
| Burke et al. (2001) (27) |  |  | ✓ |  |  |  |  |
| Chalé et al. (2013) (29) |  |  |  |  | ✓ |  |  |
| Claessens et al. (2009) (31) |  |  | ✓ |  | ✓ |  |  |
| Cribb et al. (2007) (33) |  |  | ✓ |  |  |  |  |
| Demling et al. (2000) (34) |  |  | ✓ |  | ✓ |  |  |
| Denysschen et al. (2009) (35) |  |  | ✓ | ✓ | ✓ |  |  |
| Duff et al. (2014) (37) | ✓ |  |  |  |  |  |  |
| Eliot et al. (2008) (38) |  |  | ✓ |  | ✓ |  |  |
| Fernandes et al. (2018) (40) |  |  |  |  | ✓ |  |  |
| Figueroa et al. (2013) (41) |  |  |  |  |  | ✓ |  |
| Frestedt et al. (2008) (43) |  |  |  |  |  | ✓ |  |
| Gordon et al. (2008) (45) | ✓ |  |  |  |  |  |  |
| Haidari et al. (2020) (49) |  |  |  |  | ✓ |  |  |
| Hambre et al. (2012) (50) |  |  |  |  | ✓ | ✓ |  |
| Hassanzadeh-Rostami et al. (2020) (51) |  |  |  | ✓ |  |  |  |
| Hector et al. (2015) (52) | |  |  |  | ✓ |  |  |
| Herda et al. (2013) (53) |  |  |  |  | ✓ |  |  |
| Hodgson et al. (2011) (55) |  | ✓ |  |  |  |  |  |
| Holm et al. (2008) (56) | ✓ |  |  |  |  |  |  |
| Holwerda et al. (2018) (57) |  |  |  |  | ✓ |  |  |
| Kasim-Karakas et al. (2009) (59) |  |  |  |  | ✓ |  |  |
| Keogh and Clifton (2008) (62) | ✓ |  | ✓ |  |  |  |  |
| Kerstetter et al. (2015) (63) |  |  |  |  | ✓ |  |  |
| Kirk et al. (2019) (66) |  |  |  |  | ✓ |  |  |
| Kjølbæk et al. (2017) (68) | ✓ |  |  | ✓ |  |  |  |
| Li et al. (2021) (72) |  |  |  |  | ✓ |  |  |
| Lynch et al. (2020) (74) |  |  |  |  | ✓ |  |  |
| Martens et al. (2015) (76) | ✓ |  |  |  |  |  |  |
| McAdam et al. (2018) (78) |  |  |  |  | ✓ |  |  |
| Mobley et al. (2017) (79) |  |  |  | ✓ |  |  |  |
| Moeller et al. (2003) (80) |  |  | ✓ |  |  |  |  |
| Mojtahedi et al. (2011) (82) | ✓ |  | ✓ |  | ✓ |  |  |
| Mori et al. (2018) (83) |  | ✓ |  |  | ✓ |  |  |
| Nabuco et al. (2019)a |  | ✓ |  |  | ✓ |  |  |
| Ormsbee et al. (2015) (86) |  |  |  |  | ✓ |  |  |
| Pal et al. (2010)a (88) |  |  | ✓ |  |  | ✓ |  |
| Pal et al. (2010)b (89) |  |  |  |  |  | ✓ |  |
| Pal et al (2014) (91) |  |  |  |  | ✓ |  |  |
| Piccolo et al. (2015) (93) |  |  |  |  | ✓ | ✓ |  |
| Roberson et al. (2021) (99) |  |  |  |  | ✓ |  |  |
| Sahathevan et al. (2018) (101) |  |  |  |  | ✓ |  |  |
| Sattler et al. (2008) (102) |  |  |  |  | ✓ |  |  |
| Stragier et al. (2016) (104) | ✓ |  |  |  |  |  |  |
| Stojkovic et al. (2017) (105) |  |  |  |  | ✓ |  |  |
| Sukumar et al. (2011) (107) | ✓ |  |  |  |  |  |  |
| Tahavogar et al. (2013) (108) |  |  |  |  | ✓ |  |  |
| Vatani and Golzar (2012) (112) |  |  |  |  | ✓ |  |  |
| Verreijen et al. (2015) (113) | ✓ |  |  |  | ✓ | ✓ |  |
| Volek et al. (2013) (115) |  |  |  | ✓ | ✓ |  |  |
| Weinheimer et al. (2012) (117) | ✓ |  | ✓ |  | ✓ |  |  |
| Weisgarber et al. (2012) (118) |  |  | ✓ |  |  |  |  |
| Zhu et al. (2015) (121) |  | ✓ |  |  |  |  |  |

**Supplementary Table 8 ctd.** Randomized controlled trials included among articles, and overlap among and between articles that included body weight as an outcome.

| **RCTs included among articles** | **Overlap among all articles** | **Overlap between Bergia et al. (3) & Miller et al. (7)** | **Overlap between Bergia et al. (3) & Kuo et al. (6)** | **Overlap between Bergia et al. (3) & Piri Damaghi et al. (8)** | **Overlap between Bergia et al. (3) & Sepandi et al. (10)** | **Overlap between Bergia et al. (3) & Wirunsawanya et al. (11)** | **Overlap between Kuo et al. (6) & Miller et al. (7)** |  |
| --- | --- | --- | --- | --- | --- | --- | --- | --- |
| Total number of RCTs overlapping | 0 | 3 | 0 | 2 | 3 | 3 | 0 |  |
| Adechian et al. (2012) (15) |  |  |  |  |  |  |  |  |
| Baer et al. (2011) (17) |  |  |  |  |  | x |  |  |
| Bell et al. (2017) (19) |  |  |  |  |  |  |  |  |
| Brown et al. (2020) (25) |  |  |  |  |  |  |  |  |
| Burke et al. (2001) (27) |  |  |  |  |  |  |  |  |
| Chalé et al. (2013) (29) |  |  |  |  |  |  |  |  |
| Claessens et al. (2009) (31) |  |  |  |  |  |  |  |  |
| Cribb et al. (2007) (33) |  |  |  |  |  |  |  |  |
| Demling et al. (2000) (34) |  |  |  |  |  | x |  |  |
| Denysschen et al. (2009) (35) |  |  |  |  |  |  |  |  |
| Duff et al. (2014) (37) |  |  |  |  |  |  |  |  |
| Eliot et al. (2008) (38) |  |  |  |  |  |  |  |  |
| Fernandes et al. (2018) (40) |  |  |  |  |  |  |  |  |
| Figueroa et al. (2013) (41) |  |  |  |  |  |  |  |  |
| Frestedt et al. (2008) (43) |  |  |  |  |  |  |  |  |
| Gordon et al. (2008) (45) |  |  |  |  |  |  |  |  |
| Haidari et al. (2020) (49) |  |  |  |  |  |  |  |  |
| Hambre et al. (2012) (50) |  |  |  |  |  |  |  |  |
| Hassanzadeh-Rostami et al. (2020) (51) |  |  |  |  |  |  |  |  |
| Hector et al. (2015) (52) | |  |  |  |  |  |  |  |
| Herda et al. (2013) (53) |  |  |  |  |  |  |  |  |
| Hodgson et al. (2011) (55) |  |  |  |  |  |  |  |  |
| Holm et al. (2008) (56) |  |  |  |  |  |  |  |  |
| Holwerda et al. (2018) (57) |  |  |  |  |  |  |  |  |
| Kasim-Karakas et al. (2009) (59) |  |  |  |  |  |  |  |  |
| Keogh and Clifton (2008) (62) |  | x |  |  |  |  |  |  |
| Kerstetter et al. (2015) (63) |  |  |  |  |  |  |  |  |
| Kirk et al. (2019) (66) |  |  |  |  |  |  |  |  |
| Kjølbæk et al. (2017) (68) |  |  |  | x |  |  |  |  |
| Li et al. (2021) (72) |  |  |  |  |  |  |  |  |
| Lynch et al. (2020) (74) |  |  |  |  |  |  |  |  |
| Martens et al. (2015) (76) |  |  |  |  |  |  |  |  |
| McAdam et al. (2018) (78) |  |  |  |  |  |  |  |  |
| Mobley et al. (2017) (79) |  |  |  |  |  |  |  |  |
| Moeller et al. (2003) (80) |  |  |  |  |  |  |  |  |
| Mojtahedi et al. (2011) (82) |  | x |  |  | x |  |  |  |
| Mori et al. (2018) (83) |  |  |  |  |  |  |  |  |
| Nabuco et al. (2019)a |  |  |  |  |  |  |  |  |
| Ormsbee et al. (2015) (86) |  |  |  |  |  |  |  |  |
| Pal et al. (2010)a (88) |  |  |  |  |  |  |  |  |
| Pal et al. (2010)b (89) |  |  |  |  |  |  |  |  |
| Pal et al (2014) (91) |  |  |  |  |  |  |  |  |
| Piccolo et al. (2015) (93) |  |  |  |  |  |  |  |  |
| Roberson et al. (2021) (99) |  |  |  |  |  |  |  |  |
| Sahathevan et al. (2018) (101) |  |  |  |  |  |  |  |  |
| Sattler et al. (2008) (102) |  |  |  |  |  |  |  |  |
| Stragier et al. (2016) (104) |  |  |  |  |  |  |  |  |
| Stojkovic et al. (2017) (105) |  |  |  |  |  |  |  |  |
| Sukumar et al. (2011) (107) |  |  |  |  |  |  |  |  |
| Tahavogar et al. (2013) (108) |  |  |  |  |  |  |  |  |
| Vatani and Golzar (2012) (112) |  |  |  |  |  |  |  |  |
| Verreijen et al. (2015) (113) |  |  |  |  | x | x |  |  |
| Volek et al. (2013) (115) |  |  |  |  |  |  |  |  |
| Weinheimer et al. (2012) (117) |  | x |  |  | x |  |  |  |
| Weisgarber et al. (2012) (118) |  |  |  |  |  |  |  |  |
| Zhu et al. (2015) (121) |  |  |  |  |  |  |  |  |

**Supplementary Table 8 ctd.** Randomized controlled trials included among articles, and overlap among and between articles that included body weight as an outcome.

| **RCTs included among articles** | | **Overlap between Kuo et al. (6) & Piri Damaghi et al. (8)** | | **Overlap between Kuo et al. (6) & Sepandi et al. (10)** | | **Overlap between Miller et al. (7) & Piri Damaghi et al. (8)** | | **Overlap between Miller et al. (7) & Sepandi et al. (10)** | | **Overlap between Piri Damaghi et al. (8) & Sepandi et al. (10)** | **Overlap between Piri Damaghi et al. (8) & Wirunsawanya et al. (11)** | **Overlap between Sepandi et al. (10) & Wirunsawanya et al. (11)** | |  |
| --- | --- | --- | --- | --- | --- | --- | --- | --- | --- | --- | --- | --- | --- | --- |
| Total number of RCTs overlapping | | 0 | | 1 | | 1 | | 7 | | 2 | 0 | 3 | |  |
| Adechian et al. (2012) (15) | |  | |  | |  | |  | |  |  |  | |  |
| Baer et al. (2011) (17) | |  | |  | |  | |  | |  |  |  | |  |
| Bell et al. (2017) (19) | |  | |  | |  | |  | |  |  |  | |  |
| Brown et al. (2020) (25) | |  | |  | |  | |  | |  |  |  | |  |
| Burke et al. (2001) (27) | |  | |  | |  | |  | |  |  |  | |  |
| Chalé et al. (2013) (29) | |  | |  | |  | |  | |  |  |  | |  |
| Claessens et al. (2009) (31) | |  | |  | |  | | x | |  |  |  | |  |
| Cribb et al. (2007) (33) | |  | |  | |  | |  | |  |  |  | |  |
| Demling et al. (2000) (34) | |  | |  | |  | | x | |  |  |  | |  |
| Denysschen et al. (2009) (35) | |  | |  | | x | | x | | x |  |  | |  |
| Duff et al. (2014) (37) | |  | |  | |  | |  | |  |  |  | |  |
| Eliot et al. (2008) (38) | |  | |  | |  | | x | |  |  |  | |  |
| Fernandes et al. (2018) (40) | |  | |  | |  | |  | |  |  |  | |  |
| Figueroa et al. (2013) (41) | |  | |  | |  | |  | |  |  |  | |  |
| Frestedt et al. (2008) (43) | |  | |  | |  | |  | |  |  |  | |  |
| Gordon et al. (2008) (45) | |  | |  | |  | |  | |  |  |  | |  |
| Haidari et al. (2020) (49) | |  | |  | |  | |  | |  |  |  | |  |
| Hambre et al. (2012) (50) | |  | |  | |  | |  | |  |  | x | |  |
| Hassanzadeh-Rostami et al. (2020) (51) | |  | |  | |  | |  | |  |  |  | |  |
| Hector et al. (2015) (52) | | | |  | |  | |  | |  |  |  | |  |
| Herda et al. (2013) (53) | |  | |  | |  | |  | |  |  |  | |  |
| Hodgson et al. (2011) (55) | |  | |  | |  | |  | |  |  |  | |  |
| Holm et al. (2008) (56) |  | |  | |  | |  | |  | |  |  |  |  |
| Holwerda et al. (2018) (57) | |  | |  | |  | |  | |  |  |  | |  |
| Kasim-Karakas et al. (2009) (59) | |  | |  | |  | |  | |  |  |  | |  |
| Keogh and Clifton (2008) (62) | |  | |  | |  | |  | |  |  |  | |  |
| Kerstetter et al. (2015) (63) | |  | |  | |  | |  | |  |  |  | |  |
| Kirk et al. (2019) (66) | |  | |  | |  | |  | |  |  |  | |  |
| Kjølbæk et al. (2017) (68) | |  | |  | |  | |  | |  |  |  | |  |
| Li et al. (2021) (72) | |  | |  | |  | |  | |  |  |  | |  |
| Lynch et al. (2020) (74) | |  | |  | |  | |  | |  |  |  | |  |
| Martens et al. (2015) (76) | |  | |  | |  | |  | |  |  |  | |  |
| McAdam et al. (2018) (78) | |  | |  | |  | |  | |  |  |  | |  |
| Mobley et al. (2017) (79) | |  | |  | |  | |  | |  |  |  | |  |
| Moeller et al. (2003) (80) | |  | |  | |  | |  | |  |  |  | |  |
| Mojtahedi et al. (2011) (82) | |  | |  | |  | | x | |  |  |  | |  |
| Mori et al. (2018) (83) | |  | | x | |  | |  | |  |  |  | |  |
| Nabuco et al. (2019)a | |  | | x | |  | |  | |  |  |  | |  |
| Ormsbee et al. (2015) (86) | |  | |  | |  | |  | |  |  |  | |  |
| Pal et al. (2010)a (88) | |  | |  | |  | | x | |  |  |  | |  |
| Pal et al. (2010)b (89) | |  | |  | |  | |  | |  |  |  | |  |
| Pal et al (2014) (91) | |  | |  | |  | |  | |  |  |  | |  |
| Piccolo et al. (2015) (93) | |  | |  | |  | |  | |  |  | x | |  |
| Roberson et al. (2021) (99) | |  | |  | |  | |  | |  |  |  | |  |
| Sahathevan et al. (2018) (101) | |  | |  | |  | |  | |  |  |  | |  |
| Sattler et al. (2008) (102) | |  | |  | |  | |  | |  |  |  | |  |
| Stragier et al. (2016) (104) | |  | |  | |  | |  | |  |  |  | |  |
| Stojkovic et al. (2017) (105) | |  | |  | |  | |  | |  |  |  | |  |
| Sukumar et al. (2011) (107) | |  | |  | |  | |  | |  |  |  | |  |
| Tahavogar et al. (2013) (108) | |  | |  | |  | |  | |  |  |  | |  |
| Vatani and Golzar (2012) (112) | |  | |  | |  | |  | |  |  |  | |  |
| Verreijen et al. (2015) (113) | |  | |  | |  | |  | |  |  | x | |  |
| Volek et al. (2013) (115) | |  | |  | |  | |  | | x |  |  | |  |
| Weinheimer et al. (2012) (117) | |  | |  | |  | | x | |  |  |  | |  |
| Weisgarber et al. (2012) (118) | |  | |  | |  | |  | |  |  |  | |  |
| Zhu et al. (2015) (121) | |  | |  | |  | |  | |  |  |  | |  |

RCTs, randomized controlled trials; ✓, included within review article; x, overlap among or between articles.

**Supplementary Table 9.** Randomized controlled trials included among articles and overlap

between articles that included BMI as an outcome.

| **RCTs included among articles** | **Miller et al. (2014)**  **(7)** | **Sepandi et al. (2022)**  **(10)** | **Overlap between articles** |
| --- | --- | --- | --- |
| Total number of RCTs | 2 | 17 | 1 |
| Bell et al. (2017) (19) |  | ✓ |  |
| Denysschen et al. (2009) (35) |  | ✓ |  |
| Haidari et al. (2020) (49) |  | ✓ |  |
| Hambre et al. (2012) (50) |  | ✓ |  |
| Hassanzadeh-Rostami et al. (2020) (51) | | ✓ |  |
| Holwerda et al. (2018) (57) |  | ✓ |  |
| Kasim-Karakas et al. (2009) (59) |  | ✓ |  |
| Kerstetter et al. (2015) (63) |  | ✓ |  |
| Kirk et al. (2019) (66) |  | ✓ |  |
| Mojtahedi et al. (2011) (82) | ✓ | ✓ | x |
| Ormsbee et al. (2015) (86) |  | ✓ |  |
| Pal et al. (2010)a (88) | ✓ |  |  |
| Pal et al. (2014) (91) |  | ✓ |  |
| Piccolo et al. (2015) (93) |  | ✓ |  |
| Sahathevan et al. (2018) (101) |  | ✓ |  |
| Stojkovic et al. (2017) (105) |  | ✓ |  |
| Tahavogar et al. (2014) (108) |  | ✓ |  |
| Vatani and Golzar (2012) (112) |  | ✓ |  |

RCTs, randomized controlled trials; ✓, included within review article; x, overlap among or

between articles.

**Supplementary Table 10.** Randomized controlled trials included among articles, and overlap among and between articles that included waist circumference as an outcome.

| **RCTs included among articles** | **Miller et al. (2014)**  **(7)** | **Badely et al. (2019)**  **(2)** | **Wirunsa-**  **wanya et al.**  **(2018)**  **(11)** | **Sepandi et al. (2022)**  **(10)** | **Overlap among all articles** | **Overlap between Miller et al. (7) & Badely et al. (2)** | **Overlap between Miller et al. (7) & Wirunsa-**  **wanya et al. (11)** | **Overlap between Miller et al. (7) & Sepandi et al. (10)** | **Overlap between Badely et al. (2) & Wirunsa-**  **wanya et al. (11)** | **Overlap between Badely et al. (2) & Sepandi et al. (10)** | **Overlap between Wirunsa-**  **wanya et al. (11) & Sepandi et al. (10)** |  |
| --- | --- | --- | --- | --- | --- | --- | --- | --- | --- | --- | --- | --- |
| Total number of RCTs | 3 | 18 | 5 | 10 | 0 | 1 | 1 | 5 | 3 | 2 | 0 |  |
| Arciero et al. (2016) (14) |  | ✓ |  |  |  |  |  |  |  |  |  |  |
| Beavers et al. (2015) (18) |  | ✓ |  |  |  |  |  |  |  |  |  |  |
| Bell et al. (2017) (19) |  | ✓ |  | ✓ |  |  |  |  |  | x |  |  |
| Claessens et al (2009) (31) | ✓ |  |  | ✓ |  |  |  | x |  |  |  |  |
| Denysschen et al. (2009) (35) | ✓ |  |  |  |  |  |  |  |  |  |  |  |
| Fernandes et al. (2018) (40) |  |  |  | ✓ |  |  |  |  |  |  |  |  |
| Figueroa et al. (2013) (41) |  |  | ✓ |  |  |  |  |  |  |  |  |  |
| Fluegel et al. (2010) (42) |  | ✓ |  |  |  |  |  |  |  |  |  |  |
| Fredstedt et al. (2008) (43) |  |  | ✓ |  |  |  |  |  |  |  |  |  |
| Gulati et al. (2017) (48) |  | ✓ |  |  |  |  |  |  |  |  |  |  |
| Haidari et al. (2020) (49) |  |  |  | ✓ |  |  |  |  |  |  |  |  |
| Hassanzadeh-Rostami et al. (2020) (51) | |  |  | ✓ |  |  |  |  |  |  |  |  |
| Kemmler et al. (2015) (60) |  | ✓ |  |  |  |  |  |  |  |  |  |  |
| Kemmler et al. (2018) (61) |  | ✓ |  |  |  |  |  |  |  |  |  |  |
| Kjølbæk et al. (2017) (68) |  | ✓ |  |  |  |  |  |  |  |  |  |  |
| Larsen et al. (2018) (69) |  | ✓ |  |  |  |  |  |  |  |  |  |  |
| Matsuoka et al. (2017) (77) |  | ✓ |  |  |  |  |  |  |  |  |  |  |
| Mohammadi-Sartang et al. (2018) (122) | ✓ |  |  |  |  |  |  |  |  |  |  |  |
| Nabuco et al. (2019)a (84) | |  |  |  | ✓ |  |  |  |  |  |  |  |
| Nabuco et al. (2019)b (85) |  | ✓ |  | ✓ |  |  |  | x |  |  |  |  |
| Ormsbee et al. (2015) (86) |  | ✓ |  | ✓ |  |  |  |  |  | x |  |  |
| Pal et al. (2010)a (88) | ✓ | ✓ | ✓ |  |  | x | x | x | x |  |  |  |
| Pal et al. (2014) (91) |  |  |  | ✓ |  |  |  |  |  |  |  |  |
| Piccolo et al. (2015) (93) |  | ✓ | ✓ |  |  |  |  | x |  |  |  |  |
| Reimer et al. (2017) (98) |  | ✓ |  |  |  |  |  |  |  |  |  |  |
| Tahavogar et al. (2014) (108) |  |  |  | ✓ |  |  |  |  |  |  |  |  |
| Tovar et al. (2016) (111) |  | ✓ |  |  |  |  |  |  |  |  |  |  |
| Verreijen et al. (2015) (113) |  | ✓ | ✓ |  |  |  |  | x |  |  |  |  |

RCTS, randomized controlled trials; ✓, included within review article; x, overlap among or between articles.

**Supplementary Table 11.** Randomized controlled trials included among

articles between articles that included CRP as an outcome.

| **RCTs included among articles** | **Zhou et al. (2015)**  **(13)** | **Prokopidis et al. (2022)**  **(9)** | **Overlap between articles** |
| --- | --- | --- | --- |
| Total number of RCTs | 7 | 10 | 4 |
| Bo et al. (2019) (21) |  | ✓ |  |
| Duff et al. (2014) (37) | ✓ | ✓ | x |
| Fekete et al. (2016) (39) | ✓ | ✓ | x |
| Gouni-Berthold et al. (2012) (46) | ✓ |  |  |
| Fernandes et al. (2018) (40) |  | ✓ |  |
| Kirk et al. (2021) (67) |  | ✓ |  |
| Laviolette et al. (2010) (70) | ✓ | ✓ | x |
| Nabuco et al. (2019)a (84) |  | ✓ |  |
| Pins et al. (2006) (94) | ✓ |  |  |
| Rondanelli et al. (2016) (100) |  | ✓ |  |
| Stojkovic et al. (2017) (105) |  | ✓ |  |
| Sugawara et al. (2012) (106) | ✓ |  |  |
| Weinheimer et al. (2012) (117) | ✓ | ✓ | x |

CRP, C-reactive protein; RCTs, randomized controlled trials; ✓, included

within review article; x, overlap among or between articles.

**Supplementary Table 12.** Randomized controlled trials included among articles between

articles that included hs-CRP as an outcome.

| **RCTs included among articles** | **Zhou et al. (2015)**  **(13)** | **Prokopidis et al. (2022)**  **(9)** | **Overlap between articles** |
| --- | --- | --- | --- |
| Total number of RCTs | 2 | 5 | 0 |
| Bohl et al. (2015) (22) |  | ✓ |  |
| Bumrungpert et al. (2018) (26) | | ✓ |  |
| Derosa et al. (2020) (36) |  | ✓ |  |
| Lee et al. (2007) (71) | ✓ |  |  |
| Petyaev et al. (2019) (92) | ✓ |  |  |
| Rakvaag et al. (2019) (95) |  | ✓ |  |
| Sohrabi et al. (2016) (103) |  | ✓ |  |

hs-CRP, high-sensitivity C-reactive protein; RCTs, randomized controlled trials; ✓, included

within review article.

**Supplementary Table 13.1.** AMSTAR2 analysis and ratings for the included articles

| **Author(s)** | **Q1** | **Q2** | **Q3** | **Q4** | **Q5** | **Q6** | **Q7** | **Q8** | **Q9** | **Q10** | **Q11** | **Q12** | **Q13** | **Q14** | **Q15** | **Q16** | **Quality Rating** |
| --- | --- | --- | --- | --- | --- | --- | --- | --- | --- | --- | --- | --- | --- | --- | --- | --- | --- |
| Amirani et al. (2020) (1) | Y | Y | Y | Y | Y | Y | Y | Y | Y | Y | Y | Y | Y | Y | Y | N | High |
| Badely et al. (2019) (2) | Y | Y | Y | Y | Y | Y | Y | PY | Y | N | Y | Y | N | Y | Y | N | Low |
| Bergia et al. (2018) (3) | Y | Y | Y | Y | Y | Y | Y | Y | Y | Y | Y | Y | Y | Y | Y | Y | High |
| Blair et al. (2020) (4) | Y | Y | Y | Y | Y | Y | Y | Y | Y | Y | No MA conducted | No MA conducted | Y | Y | No MA conducted | Y | High |
| Chiang et al. (2022) (5) | Y | Y | Y | Y | Y | Y | Y | Y | Y | Y | Y | Y | Y | Y | Y | Y | High |
| Kuo et al. (2022) (6) | Y | Y | Y | Y | Y | Y | Y | Y | Y | Y | Y | Y | Y | Y | Y | Y | High |
| Miller et al. (2014) (7) | Y | Y | Y | Y | Y | Y | Y | PY | PY | Y | Y | Y | Y | Y | Y | Y | High |
| Piri Damaghi et al. (2022) (8) | Y | PY | Y | PY | Y | Y | PY | Y | Y | N | Y | N | N | Y | Y | Y | Low |
| Prokopidis et al. (2022) (9) | Y | Y | Y | Y | Y | Y | Y | Y | Y | Y | Y | Y | Y | Y | Y | Y | High |
| Sepandi et al. (2022) (10) | Y | Y | Y | Y | Y | Y | Y | Y | Y | N | Y | Y | Y | Y | Y | Y | High |
| Wirunsawanya et al. (2018) (11) | Y | Y | Y | Y | Y | Y | Y | PY | Y | N | Y | Y | Y | Y | Y | N | Moderate |
| Zhang et al. (2016) (12) | Y | PY | Y | Y | N | Y | Y | Y | Y | Y | Y | Y | Y | Y | Y | Y | High |
| Zhou et al. (2015) (13) | Y | PY | Y | Y | N | N | Y | Y | Y | N | Y | Y | Y | Y | Y | N | Moderate |

N, no; PY, partial yes; Q, questions; Y, yes.

**Supplementary Table 13.2.** AMSTAR2 analysis for our umbrella systematic review of systematic reviews and meta-analyses

| **Author(s)** | **Q1** | **Q2** | **Q3** | **Q4** | **Q5** | **Q6** | **Q7** | **Q8** | **Q9** | **Q10** | **Q11** | **Q12** | **Q13** | **Q14** | **Q15** | **Q16** | **Quality Rating** |
| --- | --- | --- | --- | --- | --- | --- | --- | --- | --- | --- | --- | --- | --- | --- | --- | --- | --- |
| Connolly et al. 2023 | Y | Y | Y | Y | Y | Y | Y | Y | Y | Y | No MA conducted | No MA conducted | Y | Y | No MA conducted | Y | High |

N, no; PY, partial yes; Q, questions; Y, yes.

**Supplementary Table 14.** Subgroup analysis results from included articles.

| **Outcome** | **Author(s)** | **Subgroup analysis** | **Number of comparisons** | **WMD^a^/**  **Net change^b^** | **95% CI** | ***P-*value** |
| --- | --- | --- | --- | --- | --- | --- |
| Fasting blood glucose | Amirani et al. (1) | Participant’s age: Adults (aged 20-65 years) | 12 | −0.30^a^ | −1.29, 0.69 | NR |
|  |  | Participant’s age: Adults plus elderly aged adults (aged ≥ 20 years) | 8 | −3.79^a^ | −4.65, −2.93 | NR |
|  |  | Participants: Healthy | 12 | –2.12^a^ | –2.87, –1.37 | NR |
|  |  | Participants: Chronic disease | 8 | –2.76 ^a^ | –4.05, –1.37 | NR |
|  |  | Study duration < 12 weeks | 9 | –1.62^a^ | –2.64, –0.60 | NR |
|  |  | Study duration ≥ 12 weeks | 11 | –2.74^a^ | –3.58, –1.89 | NR |
|  |  | Type of WP: Isolated | 6 | 0.35^a^ | −1.57, 2.28 | NR |
|  |  | Type of WP: WPs | 14 | –2.72^a^ | –3.43, –2.01 | NR |
|  |  | Comparator: Placebo | 3 | −1.22^a^ | −3.33, 1.43 | NR |
|  |  | Comparator: Carbohydrates | 13 | −0.73^a^ | −1.61, 0.15 | NR |
|  |  | Comparator: Non-intervention controls | 4 | −4.54^a^ | −5.56, −3.51 | NR |
| HbA1c | Amirani et al. (1) | Participant’s age: Adults (aged 20-65 years) | 3 | –0.15^a^ | –0.21, –0.08 | NR |
|  |  | Participant’s age: Adults plus elderly aged adults (aged ≥ 20 years) | 3 | –0.09^a^ | –0.14, –0.04 | NR |
|  |  | Participants: Healthy | 3 | –0.08^a^ | –0.12, –0.03 | NR |
|  |  | Participants: Chronic disease | 3 | –0.17^a^ | –0.23, –0.10 | NR |
|  |  | Study duration < 12 weeks | 3 | –0.06^a^ | –0.10, –0.01 | NR |
|  |  | Study duration ≥ 12 weeks | 3 | –0.33^a^ | –0.42, –0.24 | NR |
| Fasting insulin | Amirani et al. (1) | Participant’s age: Adults (aged 20-65 years) | 9 | −1.43^a^ | −2.21, 0.65 | NR |
|  |  | Participant’s age: Adults plus elderly aged adults (aged ≥ 20 years) | 5 | −0.34^a^ | −0.74, 0.07 | NR |
|  |  | Participants: Healthy | 10 | –0.39^a^ | –0.78, –0.00 | NR |
|  |  | Participants: Chronic disease | 4 | –1.67^a^ | –2.63, –0.70 | NR |
|  |  | Study duration < 12 weeks | 7 | –0.49^a^ | –0.95, –0.03 | NR |
|  |  | Study duration ≥ 12 weeks | 7 | –0.69^a^ | –1.27, –0.11 | NR |
|  |  | Type of WP: Isolated | 4 | –1.15^a^ | –2.10, –0.20 | NR |
|  |  | Type of WP: WPs | 10 | –0.42^a^ | –0.81, –0.03 | NR |
|  |  | Comparator: Placebo | 2 | −3.30^a^ | −5.18, 1.42 | NR |
|  |  | Comparator: Carbohydrates | 9 | –0.64^a^ | –1.16, –0.11 | NR |
|  |  | Comparator: Non-intervention controls | 5 | −0.30^a^ | −0.81, 0.21 | NR |
| HOMA-IR | Amirani et al. (1) | Participant’s age: Adults (aged 20-65 years) | 9 | –0.25^a^ | –0.38, –0.11 | NR |
|  |  | Participant’s age: Adults plus elderly aged adults (aged ≥ 20 years) | 4 | –0.07^a^ | –0.13, –0.01 | NR |
|  |  | Participants: Healthy | 8 | –0.07^a^ | –0.13, –0.01 | NR |
|  |  | Participants: Chronic disease | 5 | –0.48^a^ | –0.70, –0.26 | NR |
|  |  | Study duration < 12 weeks | 7 | −0.04^a^ | −0.14, 0.06 | NR |
|  |  | Study duration ≥ 12 weeks | 6 | −0.12^a^ | −0.19, − 0.06 | NR |
|  |  | Type of WP: Isolated | 4 | −0.20^a^ | −0.33, − 0.06 | NR |
|  |  | Type of WP: WPs | 9 | −0.07^a^ | 0.13, − 0.01 | NR |
|  |  | Comparator: Placebo | 2 | −0.86^a^ | −1.43, − 0.29 | NR |
|  |  | Comparator: Carbohydrates | 8 | −0.22^a^ | −0.35, − 0.10 | NR |
|  |  | Comparator: Non-intervention controls | 3 | −0.06^a^ | −0.12, 0.01 | NR |
| Total cholesterol | Amirani et al. (1) | Participant’s age: Adults (aged 20-65 years) | 12 | −12.86^a^ | −16.11, −9.61 | NR |
|  |  | Participant’s age: Adults plus elderly aged adults (aged ≥ 20 years) | 10 | −9.07^a^ | −11.39, −6.74 | NR |
|  |  | Participants: Healthy | 15 | −8.75^a^ | −10.87, −6.63 | NR |
|  |  | Participants: Chronic disease | 7 | −16.40^a^ | −20.53, −12.27 | NR |
|  |  | Study duration < 12 weeks | 10 | −15.83^a^ | −18.33, −13.34 | NR |
|  |  | Study duration ≥ 12 weeks | 12 | −3.01^a^ | −5.90, −0.12 | NR |
|  |  | Type of WP: Isolated | 7 | −9.67^a^ | −12.52, −6.82 | NR |
|  |  | Type of WP: WPs | 15 | − 11.77^a^ | −14.43, −9.11 | NR |
|  |  | Comparator: Placebo | 2 | −1.99^a^ | −10.44, 6.47 | NR |
|  |  | Comparator: Carbohydrates | 16 | −11.49^a^ | −13.76, −9.22 | NR |
|  |  | Comparator: Non-intervention controls | 4 | −8.92^a^ | −12.63, −5.20 | NR |
| LDL-C | Amirani et al. (1) | Participant’s age: Adults (aged 20-65 years) | 9 | −1.73^a^ | −5.32, 1.87 | NR |
|  |  | Participant’s age: Adults plus elderly aged adults (aged ≥ 20 years) | 10 | −8.90^a^ | −10.98, −6.81 | NR |
|  |  | Participants: Healthy | 14 | −8.31^a^ | −10.25, −6.36 | NR |
|  |  | Participants: Chronic disease | 5 | 0.45^a^ | −4.39, 5.29 | NR |
|  |  | Study duration < 12 weeks | 8 | −18.51^a^ | −21.39, −15.62 | NR |
|  |  | Study duration ≥ 12 weeks | 11 | 0.22^a^ | −2.09, 2.52 | NR |
|  |  | Type of WP: Isolated | 5 | −10.49^a^ | −13.75, −7.22 | NR |
|  |  | Type of WP: WPs | 14 | −6.41^a^ | −8.67, −4.15 | NR |
|  |  | Comparator: Placebo | 2 | 4.36^a^ | −2.84, 11.56 | NR |
|  |  | Comparator: Carbohydrates | 14 | −7.75^a^ | −10.28, 5.23 | NR |
|  |  | Comparator: Non-intervention controls | 3 | −7.98^a^ | −10.74, −5.22 | NR |
| HDL-C | Amirani et al. (1) | Participant’s age: Adults (aged 20-65 years) | 11 | −1.65^a^ | −2.41, −0.89 | NR |
|  |  | Participant’s age: Adults plus elderly aged adults (aged ≥ 20 years) | 10 | 1.40^a^ | 1.10, 1.71 | NR |
|  |  | Participants: Healthy | 14 | 1.48^a^ | 1.17, 1.79 | NR |
|  |  | Participants: Chronic disease | 7 | −1.94^a^ | −2.68, −1.20 | NR |
|  |  | Study duration < 12 weeks | 9 | 1.05^a^ | 0.76, 1.35 | NR |
|  |  | Study duration ≥ 12 weeks | 12 | 0.32^a^ | −0.58, 1.22 | NR |
|  |  | Type of WP: Isolated | 6 | 1.57^a^ | 1.25, 1.89 | NR |
|  |  | Type of WP: WPs | 15 | −1.15^a^ | −1.79, −0.51 | NR |
|  |  | Comparator: Placebo | 2 | 1.61^a^ | −0.09, 3.32 | NR |
|  |  | Comparator: Carbohydrates | 16 | 1.04^a^ | 0.74, 1.33 | NR |
|  |  | Comparator: Non-intervention controls | 3 | 0.03^a^ | −1.03, 1.09 | NR |
| Triglycerides | Amirani et al. (1) | Participant’s age: Adults (aged 20-65 years) | 11 | −6.78^a^ | −10.71, −2.85 | NR |
|  |  | Participant’s age: Adults plus elderly aged adults (aged ≥ 20 years) | 11 | −21.43^a^ | − 24.28, −18.58 | NR |
|  |  | Participants: Healthy | 15 | −15.58^a^ | − 17.99, −13.16 | NR |
|  |  | Participants: Chronic disease | 7 | −25.04^a^ | −32.96, −17.11 | NR |
|  |  | Study duration < 12 weeks | 9 | −15.63^a^ | −18.60, −12.66 | NR |
|  |  | Study duration ≥ 12 weeks | 13 | −17.52^a^ | −21.19, −13.85 | NR |
|  |  | Type of WP: Isolated | 7 | −13.90^a^ | −16.94, 10.87 | NR |
|  |  | Type of WP: WPs | 15 | −19.86^a^ | −23.47, −16.25 | NR |
|  |  | Comparator: Placebo | 2 | −25.36^a^ | −41.44, −0.29 | NR |
|  |  | Comparator: Carbohydrates | 16 | −15.14^a^ | −17.90, −12.38 | NR |
|  |  | Comparator: Non-intervention controls | 4 | −18.81^a^ | −23.18, −14.44 | NR |
|  | Zhang et al. (12) | BMI ≥ 30 kg/m^2^ | 7 | −0.18 mmo/L^b^ | −0.35, −0.01 mmol/L | 0.04 |
|  |  | BMI < 30 kg/m^2^ | 6 | − 0.05 mmol/L^b^ | −0.19, 0.08 mmol/L | 0.45 |
|  |  | Baseline LDL-C ≥ 3.2 mmol/L | 5 | −0.09 mmo/L^b^ | −0.23, 0.03 mmo/L | 0.17 |
|  |  | Baseline LDL-C < 3.2 mmol/L | 6 | −0.12 mmo/L^b^ | −0.31, 0.08 mmo/L | 0.23 |
|  |  | WP dose ≥ 30 g/d | 6 | −0.21 mmol/L^b^ | −0.40, −0.02 mmol/L | 0.03 |
|  |  | WP dose < 30 g/d | 6 | − 0.11 mmo/L^b^ | −0.21, 0.01 mmo/L | 0.14 |
|  |  | Exercise training or in energy restriction: No | 7 | − 0.16 mmo/L^b^ | −0.31, 0 mmo/L | 0.05 |
|  |  | Exercise training or in energy restriction: Yes | 6 | −0.06 mmo/L^b^ | −0.21, 0.08 mmo/L | 0.40 |
| Body weight | Bergia et al. (3) | Without energy restriction and with resistance training | 6 | −0.23 kg^a^ | −1.41, 0.96 kg | NR |
|  |  | Without energy restriction and with resistance training | 3 | −0.12 kg^a^ | −1.23, 0.99 kg | NR |
|  |  | With energy restriction and without resistance training | 5 | 0.48 kg^a^ | −0.51, 0.47 kg | NR |
|  | Kuo et al. (6) | Without resistance training | 2 | −0.1435^a^ | −0.344, 0.057 | NR |
|  |  | With resistance training | 2 | −0.0205^a^ | −0.386, 0.345 | NR |
|  | Miller et al. (7) | Without resistance training | 2 | −2.66 kg^a^ | −6.16, 0.84 kg | NR |
|  |  | With resistance exercise | 6 | −0.02 kg^a^ | −1.42, 1.37 kg | NR |
|  |  | WP isolate | NR | −2.23 kg^a^ | −6.49, 2.03 kg | NR |
|  |  | WP concentrate | NR | −0.16 kg^a^ | −1.67, 1.35 kg | NR |
|  | Piri Damaghi et al. (8) | With exercise | 3 | 0·93^a^ | −0.62, 2.49 | 0·239 |
|  |  | Without exercise | 3 | −1.37^a^ | −2.33, −0.41 | 0.005 |
|  |  | WP dose < 50 g/d | 2 | −0.25^a^ | −2.06, 1.56 | 0.788 |
|  |  | WP dose > 50 g/d | 3 | −0.50^a^ | −2.75, 1.75 | 0.663 |
|  |  | Study duration < 12 weeks | 1 | −0.10^a^ | −8.26, 8.06 | 0.981 |
|  |  | Study duration > 12 weeks | 5 | −0.46^a^ | −2.04, 1.11 | 0.566 |
|  |  | Health status: Healthy | 2 | 0.91^a^ | −1.04, 2.86 | 0.361 |
|  |  | Health status: Overweight/obese | 3 | −1.37^a^ | −2.33, −0.41 | 0.005 |
|  |  | Sex: Male | 2 | −1.24^a^ | −5.34, 2.86 | 0.553 |
|  |  | Sex: Female + Male | 4 | −0.32^a^ | −2.11, 1.46 | 0.721 |
| CRP | Zhou et al. (13) | Study duration < 12 weeks | 4 | −0.60 mg/L^b^ | −1.47, 0.26 mg/L | 0.17 |
|  |  | Study duration ≥ 12 weeks | 5 | −0.30 mg/L^b^ | −1.02, 0.42 mg/L | 0.41 |
|  |  | WP dose < 20 g/d | 4 | −0.10 mg/L^b^ | −0.69, 0.49 mg/L | 0.74 |
|  |  | WP dose ≥ 20 g/d | 5 | −0.72 mg/L^b^ | −0.97, −0.47 mg/L | <0.01 |
|  |  | Baseline CRP < 3 mg/L | 5 | −0.06 mg/L^b^ | −0.46, 0.35 mg/L | 0.79 |
|  |  | Baseline CRP ≥ 3 mg/L | 4 | −0.67 mg/L^b^ | −1.21, −0.14 mg/L | 0.01 |
|  | Prokopidis et al. (9) | Participants aged < 60 y | 3 | −0.19 mg/L^a^ | −0.61, 0.23 mg/L | 0.38 |
|  |  | Participants aged ≥ 60 y | 7 | −0.07 mg/L^a^ | −0.52, 0.39 mg/L | 0.77 |
|  |  | Participants classified as with sarcopenia or pre-frailty | 3 | 0.02 mg/L^a^ | −1.60, 1.65 mg/L | 0.98 |
|  |  | Study duration ≤ 8 weeks | 3 | −0.29 mg/L^a^ | −0.38, −0.20 mg/L | < 0.00001 |
|  |  | Study duration > 8 weeks | 7 | 0.01 mg/L^a^ | −0.41, 0.43 mg/L | 0.95 |
|  |  | BMI < 25 kg/m^2^ | 2 | −0.65 mg/L^a^ | −1.23, −0.06 mg/L | 0.03 |
|  |  | BMI ≥ 25 kg/m^2^ | 8 | 0.00 mg/L^a^ | −0.32, 0.32 mg/L | 0.98 |
|  |  | WP dose < 30 g/d | 2 | −1.24 mg/L^a^ | −4.28, 1.79 mg/L | 0.42 |
|  |  | WP dose ≥ 30 g/d | 6 | −0.04 mg/L^a^ | −0.08, 0.00 mg/L | 0.07 |
| hs-CRP | Prokopidis et al. (9) | Participants aged < 60 y | 3 | 0.05 mg/L^a^ | −0.79, 0.90 mg/L | 0.90 |
|  |  | Participants aged ≥ 60 y | 3 | 0.27 mg/L^a^ | −0.22, 0.77 mg/L | 0.67 |
|  |  | BMI < 25 kg/m^2^ | 3 | 0.05 mg/L^a^ | −0.79, 0.90 mg/L | 0.90 |
|  |  | BMI ≥ 25 kg/m^2^ | 2 | 0.27 mg/L^a^ | −0.22, 0.77 mg/L | 0.28 |
|  |  | WP dose < 30 g/d | 3 | −0.29 mg/L^a^ | −1.01, 0.43 mg/L | 0.43 |
|  |  | WP dose ≥ 30 g/d | 7 | 0.03 mg/L^a^ | −0.43, 0.49 mg/L | 0.91 |

BMI, body mass index; CRP, C-reactive protein; HbA1c, hemoglobin A1c; HDL-C, high-density lipoprotein cholesterol; HOMA-IR, Homeostatic Model Assessment for Insulin Resistance; hs-CRP, high sensitivity C-reactive protein; LDL-C, low-density lipoprotein cholesterol; NR, not reported; WMD, weighted mean difference; WP, whey protein; y, years.

**Supplementary Table 15.** GRADE approach ratings for certainty of evidence included among articles.

|  | **Amirani et al.**  **(2018) (1)** | **Chiang et al.**  **(2022) (5)** | **Kuo et al.**  **(2022) (6)** | **Prokipidis et al.**  **(2022) (9)** |
| --- | --- | --- | --- | --- |
| **GRADE approach ratings for certainty of evidence for outcomes** | ↔ fasting blood glucose (n = 20): Low  ↓ fasting insulin (n = 14): Moderate  ↓ HbA1c (n = 6): Moderate  ↓ HOMA-IR (n = 13): Low  ↓ Total cholesterol (n = 22): Low  ↓ LDL-C (n = 19): Low  ↔ HDL-C (n = 21): Very low  ↓ TG (n = 22): Low | ↓ postprandial glucose at 60 min (n = 5): Low  ↓ postprandial glucose at 120 min (n = 4): Low  ↔ in postprandial insulin at 60 min (n = 3): Very low  ↔ in postprandial insulin at 120 min (n = 3): Very low  ↔ glucose iAUC (n = 2): Very low  ↑ insulin iAUC (n=3): Low | ↔ in body weight:  Combined WP with and without RT (n = 4): Moderate  WP with RT (n = 2): Low  WP without RT (n = 2): Moderate | ↔ in CRP (n = 10): Moderate  ↔ in hs-CRP (n = 5): High |

CRP, C-reactive protein; GRADE, Grading of Recommendations Assessment, Development and Evaluation; HbA1c, hemoglobin A1c; HDL-C, high-density lipoprotein cholesterol; HOMA-IR, Homesostatic Model Assessment for Insulin Resistance; hs-CRP, high-sensitivity C-reactive protein; iAUC, incremental area under the curve; LDL-C, low-density lipoprotein cholesterol; RT, resistance training; TG, triglycerdies; WP, whey protein.

**Supplementary references**

1. Amirani E, Milajerdi A, Reiner Ž, Mirzaei H, Mansournia MA, Asemi Z. Effects of whey protein on glycemic control and serum lipoproteins in patients with metabolic syndrome and related conditions: a systematic review and meta-analysis of randomized controlled clinical trials. Lipids Health Dis 2020;19(1):209. doi: 10.1186/s12944-020-01384-7.

2. Badely M, Sepandi M, Samadi M, Parastouei K, Taghdir M. The effect of whey protein on the components of metabolic syndrome in overweight and obese individuals; a systematic review and meta-analysis. Diabetes Metab Syndr 2019;13(6):3121-31. doi: 10.1016/j.dsx.2019.11.001.

3. Bergia RE, 3rd, Hudson JL, Campbell WW. Effect of whey protein supplementation on body composition changes in women: a systematic review and meta-analysis. Nutr Rev 2018;76(7):539-51. doi: 10.1093/nutrit/nuy017.

4. Blair M, Kellow NJ, Dordevic AL, Evans S, Caissutti J, McCaffrey TA. Health Benefits of Whey or Colostrum Supplementation in Adults ≥35 Years; a Systematic Review. Nutrients 2020;12(2). doi: 10.3390/nu12020299.

5. Chiang SW, Liu HW, Loh EW, Tam KW, Wang JY, Huang WL, Kuan YC. Whey protein supplementation improves postprandial glycemia in persons with type 2 diabetes mellitus: A systematic review and meta-analysis of randomized controlled trials. Nutr Res 2022;104:44-54. doi: 10.1016/j.nutres.2022.04.002.

6. Kuo Y-Y, Chang H-Y, Huang Y-C, Liu C-W. Effect of Whey Protein Supplementation in Postmenopausal Women: A Systematic Review and Meta-Analysis. Nutrients 2022;14(19):4210.

7. Miller PE, Alexander DD, Perez V. Effects of whey protein and resistance exercise on body composition: a meta-analysis of randomized controlled trials. J Am Coll Nutr 2014;33(2):163-75. doi: 10.1080/07315724.2013.875365.

8. Piri Damaghi M, Mirzababaei A, Moradi S, Daneshzad E, Tavakoli A, Clark CCT, Mirzaei K. Comparison of the effect of soya protein and whey protein on body composition: a meta-analysis of randomised clinical trials. Br J Nutr 2022;127(6):885-95. doi: 10.1017/s0007114521001550.

9. Prokopidis K, Mazidi M, Sankaranarayanan R, Tajik B, McArdle A, Isanejad M. Effects of whey and soy protein supplementation on inflammatory cytokines in older adults: a systematic review and meta-analysis. Br J Nutr 2022:1-12. doi: 10.1017/s0007114522001787.

10. Sepandi M, Samadi M, Shirvani H, Alimohamadi Y, Taghdir M, Goudarzi F, Akbarzadeh I. Effect of whey protein supplementation on weight and body composition indicators: A meta-analysis of randomized clinical trials. Clin Nutr ESPEN 2022;50:74-83. doi: 10.1016/j.clnesp.2022.05.020.

11. Wirunsawanya K, Upala S, Jaruvongvanich V, Sanguankeo A. Whey Protein Supplementation Improves Body Composition and Cardiovascular Risk Factors in Overweight and Obese Patients: A Systematic Review and Meta-Analysis. J Am Coll Nutr 2018;37(1):60-70. doi: 10.1080/07315724.2017.1344591.

12. Zhang JW, Tong X, Wan Z, Wang Y, Qin LQ, Szeto IM. Effect of whey protein on blood lipid profiles: a meta-analysis of randomized controlled trials. Eur J Clin Nutr 2016;70(8):879-85. doi: 10.1038/ejcn.2016.39.

13. Zhou LM, Xu JY, Rao CP, Han S, Wan Z, Qin LQ. Effect of whey supplementation on circulating C-reactive protein: a meta-analysis of randomized controlled trials. Nutrients 2015;7(2):1131-43. doi: 10.3390/nu7021131.

14. Arciero PJ, Edmonds RC, Bunsawat K, Gentile CL, Ketcham C, Darin C, Renna M, Zheng Q, Zhang JZ, Ormsbee MJ. Protein-Pacing from Food or Supplementation Improves Physical Performance in Overweight Men and Women: The PRISE 2 Study. Nutrients 2016;8(5). doi: 10.3390/nu8050288.

15. Adechian S, Balage M, Remond D, Migné C, Quignard-Boulangé A, Marset-Baglieri A, Rousset S, Boirie Y, Gaudichon C, Dardevet D, et al. Protein feeding pattern, casein feeding, or milk-soluble protein feeding did not change the evolution of body composition during a short-term weight loss program. Am J Physiol Endocrinol Metab 2012;303(8):E973-82. doi: 10.1152/ajpendo.00285.2012.

16. Aldrich ND, Reicks MM, Sibley SD, Redmon JB, Thomas W, Raatz SK. Varying protein source and quantity do not significantly improve weight loss, fat loss, or satiety in reduced energy diets among midlife adults. Nutr Res 2011;31(2):104-12. doi: 10.1016/j.nutres.2011.01.004.

17. Baer DJ, Stote KS, Paul DR, Harris GK, Rumpler WV, Clevidence BA. Whey protein but not soy protein supplementation alters body weight and composition in free-living overweight and obese adults. J Nutr 2011;141(8):1489-94. doi: 10.3945/jn.111.139840.

18. Beavers KM, Gordon MM, Easter L, Beavers DP, Hairston KG, Nicklas BJ, Vitolins MZ. Effect of protein source during weight loss on body composition, cardiometabolic risk and physical performance in abdominally obese, older adults: a pilot feeding study. J Nutr Health Aging 2015;19(1):87-95. doi: 10.1007/s12603-015-0438-7.

19. Bell KE, Snijders T, Zulyniak M, Kumbhare D, Parise G, Chabowski A, Phillips SM. A whey protein-based multi-ingredient nutritional supplement stimulates gains in lean body mass and strength in healthy older men: A randomized controlled trial. PLoS One 2017;12(7):e0181387. doi: 10.1371/journal.pone.0181387.

20. Berthold HK, Schulte DM, Lapointe JF, Lemieux P, Krone W, Gouni-Berthold I. The whey fermentation product malleable protein matrix decreases triglyceride concentrations in subjects with hypercholesterolemia: a randomized placebo-controlled trial. J Dairy Sci 2011;94(2):589-601. doi: 10.3168/jds.2010-3115.

21. Bo Y, Liu C, Ji Z, Yang R, An Q, Zhang X, You J, Duan D, Sun Y, Zhu Y, et al. A high whey protein, vitamin D and E supplement preserves muscle mass, strength, and quality of life in sarcopenic older adults: A double-blind randomized controlled trial. Clin Nutr 2019;38(1):159-64. doi: 10.1016/j.clnu.2017.12.020.

22. Bohl M, Bjørnshave A, Gregersen S, Hermansen K. Whey and Casein Proteins and Medium-Chain Saturated Fatty Acids from Milk Do Not Increase Low-Grade Inflammation in Abdominally Obese Adults. Rev Diabet Stud 2016;13(2-3):148-57. doi: 10.1900/rds.2016.13.148.

23. Bohl M, Bjørnshave A, Larsen MK, Gregersen S, Hermansen K. The effects of proteins and medium-chain fatty acids from milk on body composition, insulin sensitivity and blood pressure in abdominally obese adults. Eur J Clin Nutr 2017;71(1):76-82. doi: 10.1038/ejcn.2016.207.

24. Brown EC, DiSilvestro RA, Babaknia A, Devor ST. Soy versus whey protein bars: effects on exercise training impact on lean body mass and antioxidant status. Nutr J 2004;3:22. doi: 10.1186/1475-2891-3-22.

25. Brown AF, Welsh T, Panton LB, Moffatt RJ, Ormsbee MJ. Higher-protein intake improves body composition index in female collegiate dancers. Applied Physiology, Nutrition, and Metabolism 2020;45(5):547-54. doi: 10.1139/apnm-2019-0517 %M 31647886.

26. Bumrungpert A, Pavadhgul P, Nunthanawanich P, Sirikanchanarod A, Adulbhan A. Whey Protein Supplementation Improves Nutritional Status, Glutathione Levels, and Immune Function in Cancer Patients: A Randomized, Double-Blind Controlled Trial. J Med Food 2018;21(6):612-6. doi: 10.1089/jmf.2017.4080.

27. Burke DG, Chilibeck PD, Davidson KS, Candow DG, Farthing J, Smith-Palmer T. The effect of whey protein supplementation with and without creatine monohydrate combined with resistance training on lean tissue mass and muscle strength. Int J Sport Nutr Exerc Metab 2001;11(3):349-64. doi: 10.1123/ijsnem.11.3.349.

28. Candow DG, Burke NC, Smith-Palmer T, Burke DG. Effect of whey and soy protein supplementation combined with resistance training in young adults. Int J Sport Nutr Exerc Metab 2006;16(3):233-44. doi: 10.1123/ijsnem.16.3.233.

29. Chalé A, Cloutier GJ, Hau C, Phillips EM, Dallal GE, Fielding RA. Efficacy of whey protein supplementation on resistance exercise-induced changes in lean mass, muscle strength, and physical function in mobility-limited older adults. J Gerontol A Biol Sci Med Sci 2013;68(6):682-90. doi: 10.1093/gerona/gls221.

30. Chiu S, Williams PT, Dawson T, Bergman RN, Stefanovski D, Watkins SM, Krauss RM. Diets high in protein or saturated fat do not affect insulin sensitivity or plasma concentrations of lipids and lipoproteins in overweight and obese adults. J Nutr 2014;144(11):1753-9. doi: 10.3945/jn.114.197624.

31. Claessens M, van Baak MA, Monsheimer S, Saris WH. The effect of a low-fat, high-protein or high-carbohydrate ad libitum diet on weight loss maintenance and metabolic risk factors. Int J Obes (Lond) 2009;33(3):296-304. doi: 10.1038/ijo.2008.278.

32. Cribb PJ, Williams AD, Carey MF, Hayes A. The effect of whey isolate and resistance training on strength, body composition, and plasma glutamine. Int J Sport Nutr Exerc Metab 2006;16(5):494-509. doi: 10.1123/ijsnem.16.5.494.

33. Cribb PJ, Williams AD, Stathis CG, Carey MF, Hayes A. Effects of whey isolate, creatine, and resistance training on muscle hypertrophy. Med Sci Sports Exerc 2007;39(2):298-307. doi: 10.1249/01.mss.0000247002.32589.ef.

34. Demling RH, DeSanti L. Effect of a hypocaloric diet, increased protein intake and resistance training on lean mass gains and fat mass loss in overweight police officers. Ann Nutr Metab 2000;44(1):21-9. doi: 10.1159/000012817.

35. Denysschen CA, Burton HW, Horvath PJ, Leddy JJ, Browne RW. Resistance training with soy vs whey protein supplements in hyperlipidemic males. J Int Soc Sports Nutr 2009;6:8. doi: 10.1186/1550-2783-6-8.

36. Derosa G, D'Angelo A, Maffioli P. Change of some oxidative stress parameters after supplementation with whey protein isolate in patients with type 2 diabetes. Nutrition 2020;73:110700. doi: 10.1016/j.nut.2019.110700.

37. Duff WR, Chilibeck PD, Rooke JJ, Kaviani M, Krentz JR, Haines DM. The effect of bovine colostrum supplementation in older adults during resistance training. Int J Sport Nutr Exerc Metab 2014;24(3):276-85. doi: 10.1123/ijsnem.2013-0182.

38. Eliot KA, Knehans AW, Bemben DA, Witten MS, Carter J, Bemben MG. The effects of creatine and whey protein supplementation on body composition in men aged 48 to 72 years during resistance training. J Nutr Health Aging 2008;12(3):208-12. doi: 10.1007/bf02982622.

39. Fekete Á A, Giromini C, Chatzidiakou Y, Givens DI, Lovegrove JA. Whey protein lowers blood pressure and improves endothelial function and lipid biomarkers in adults with prehypertension and mild hypertension: results from the chronic Whey2Go randomized controlled trial. Am J Clin Nutr 2016;104(6):1534-44. doi: 10.3945/ajcn.116.137919.

40. Fernandes RR, Nabuco HCG, Sugihara Junior P, Cavalcante EF, Fabro PMC, Tomeleri CM, Ribeiro AS, Barbosa DS, Venturini D, Schoenfeld BJ, et al. Effect of protein intake beyond habitual intakes following resistance training on cardiometabolic risk disease parameters in pre-conditioned older women. Exp Gerontol 2018;110:9-14. doi: 10.1016/j.exger.2018.05.003.

41. Figueroa A, Wong A, Kinsey A, Kalfon R, Eddy W, Ormsbee MJ. Effects of milk proteins and combined exercise training on aortic hemodynamics and arterial stiffness in young obese women with high blood pressure. Am J Hypertens 2014;27(3):338-44. doi: 10.1093/ajh/hpt224.

42. Fluegel SM, Shultz TD, Powers JR, Clark S, Barbosa-Leiker C, Wright BR, Freson TS, Fluegel HA, Minch JD, Schwarzkopf LK, et al. Whey beverages decrease blood pressure in prehypertensive and hypertensive young men and women. International Dairy Journal 2010;20(11):753-60. doi: <https://doi.org/10.1016/j.idairyj.2010.06.005>.

43. Frestedt JL, Zenk JL, Kuskowski MA, Ward LS, Bastian ED. A whey-protein supplement increases fat loss and spares lean muscle in obese subjects: a randomized human clinical study. Nutr Metab (Lond) 2008;5:8. doi: 10.1186/1743-7075-5-8.

44. Gaffney KA, Lucero A, Stoner L, Faulkner J, Whitfield P, Krebs J, Rowlands DS. Nil Whey Protein Effect on Glycemic Control after Intense Mixed-Mode Training in Type 2 Diabetes. Med Sci Sports Exerc 2018;50(1):11-7. doi: 10.1249/mss.0000000000001404.

45. Gordon MM, Bopp MJ, Easter L, Miller GD, Lyles MF, Houston DK, Nicklas BJ, Kritchevsky SB. Effects of dietary protein on the composition of weight loss in post-menopausal women. J Nutr Health Aging 2008;12(8):505-9. doi: 10.1007/bf02983202.

46. Gouni-Berthold I, Schulte DM, Krone W, Lapointe JF, Lemieux P, Predel HG, Berthold HK. The whey fermentation product malleable protein matrix decreases TAG concentrations in patients with the metabolic syndrome: a randomised placebo-controlled trial. Br J Nutr 2012;107(11):1694-706. doi: 10.1017/s0007114511004843.

47. Gryson C, Ratel S, Rance M, Penando S, Bonhomme C, Le Ruyet P, Duclos M, Boirie Y, Walrand S. Four-month course of soluble milk proteins interacts with exercise to improve muscle strength and delay fatigue in elderly participants. J Am Med Dir Assoc 2014;15(12):958.e1-9. doi: 10.1016/j.jamda.2014.09.011.

48. Gulati S, Misra A, Tiwari R, Sharma M, Pandey RM, Yadav CP. Effect of high-protein meal replacement on weight and cardiometabolic profile in overweight/obese Asian Indians in North India. Br J Nutr 2017;117(11):1531-40. doi: 10.1017/s0007114517001295.

49. Haidari F, Aghamohammadi V, Mohammadshahi M, Ahmadi-Angali K, Asghari-Jafarabadi M. Whey protein supplementation reducing fasting levels of anandamide and 2-AG without weight loss in pre-menopausal women with obesity on a weight-loss diet. Trials 2020;21(1):657. doi: 10.1186/s13063-020-04586-7.

50. Hambre D, Vergara M, Lood Y, Bachrach-Lindström M, Lindström T, Nystrom FH. A randomized trial of protein supplementation compared with extra fast food on the effects of resistance training to increase metabolism. Scand J Clin Lab Invest 2012;72(6):471-8. doi: 10.3109/00365513.2012.698021.

51. Hassanzadeh-Rostami Z, Abbasi A, Faghih S. Effects of biscuit fortified with whey protein isolate and wheat bran on weight loss, energy intake, appetite score, and appetite regulating hormones among overweight or obese adults. Journal of Functional Foods 2020;70:103743. doi: <https://doi.org/10.1016/j.jff.2019.103743>.

52. Hector AJ, Marcotte GR, Churchward-Venne TA, Murphy CH, Breen L, von Allmen M, Baker SK, Phillips SM. Whey protein supplementation preserves postprandial myofibrillar protein synthesis during short-term energy restriction in overweight and obese adults. J Nutr 2015;145(2):246-52. doi: 10.3945/jn.114.200832.

53. Herda AA, Herda TJ, Costa PB, Ryan ED, Stout JR, Cramer JT. Muscle performance, size, and safety responses after eight weeks of resistance training and protein supplementation: a randomized, double-blinded, placebo-controlled clinical trial. J Strength Cond Res 2013;27(11):3091-100. doi: 10.1519/JSC.0b013e31828c289f.

54. Herda AA, McKay BD, Herda TJ, Costa PB, Stout JR, Cramer JT. Changes in Strength, Mobility, and Body Composition Following Self-Selected Exercise in Older Adults. J Aging Phys Act 2021;29(1):17-26. doi: 10.1123/japa.2019-0468.

55. Hodgson JM, Zhu K, Lewis JR, Kerr D, Meng X, Solah V, Devine A, Binns CW, Woodman RJ, Prince RL. Long-term effects of a protein-enriched diet on blood pressure in older women. Br J Nutr 2012;107(11):1664-72. doi: 10.1017/s0007114511004740.

56. Holm L, Olesen JL, Matsumoto K, Doi T, Mizuno M, Alsted TJ, Mackey AL, Schwarz P, Kjaer M. Protein-containing nutrient supplementation following strength training enhances the effect on muscle mass, strength, and bone formation in postmenopausal women. J Appl Physiol (1985) 2008;105(1):274-81. doi: 10.1152/japplphysiol.00935.2007.

57. Holwerda AM, Overkamp M, Paulussen KJM, Smeets JSJ, van Kranenburg J, Backx EMP, Gijsen AP, Goessens JPB, Verdijk LB, van Loon LJC. Protein Supplementation after Exercise and before Sleep Does Not Further Augment Muscle Mass and Strength Gains during Resistance Exercise Training in Active Older Men. J Nutr 2018;148(11):1723-32. doi: 10.1093/jn/nxy169.

58. Jakubowicz D, Wainstein J, Landau Z, Ahren B, Barnea M, Bar-Dayan Y, Froy O. High-energy breakfast based on whey protein reduces body weight, postprandial glycemia and HbA(1C) in Type 2 diabetes. J Nutr Biochem 2017;49:1-7. doi: 10.1016/j.jnutbio.2017.07.005.

59. Kasim-Karakas SE, Almario RU, Cunningham W. Effects of protein versus simple sugar intake on weight loss in polycystic ovary syndrome (according to the National Institutes of Health criteria). Fertil Steril 2009;92(1):262-70. doi: 10.1016/j.fertnstert.2008.05.065.

60. Kemmler W, Wittke A, Bebenek M, Fröhlich M, von Stengel S. High Intensity Resistance Training Methods with and without Protein Supplementation to Fight Cardiometabolic Risk in Middle-Aged Males: A Randomized Controlled Trial. Biomed Res Int 2016;2016:9705287. doi: 10.1155/2016/9705287.

61. Kemmler W, Kohl M, Freiberger E, Sieber C, von Stengel S. Effect of whole-body electromyostimulation and / or protein supplementation on obesity and cardiometabolic risk in older men with sarcopenic obesity: the randomized controlled FranSO trial. BMC Geriatr 2018;18(1):70. doi: 10.1186/s12877-018-0759-6.

62. Keogh JB, Clifton P. The effect of meal replacements high in glycomacropeptide on weight loss and markers of cardiovascular disease risk. Am J Clin Nutr 2008;87(6):1602-5. doi: 10.1093/ajcn/87.6.1602.

63. Kerstetter JE, Bihuniak JD, Brindisi J, Sullivan RR, Mangano KM, Larocque S, Kotler BM, Simpson CA, Cusano AM, Gaffney-Stomberg E, et al. The Effect of a Whey Protein Supplement on Bone Mass in Older Caucasian Adults. J Clin Endocrinol Metab 2015;100(6):2214-22. doi: 10.1210/jc.2014-3792.

64. King DG, Walker M, Campbell MD, Breen L, Stevenson EJ, West DJ. A small dose of whey protein co-ingested with mixed-macronutrient breakfast and lunch meals improves postprandial glycemia and suppresses appetite in men with type 2 diabetes: a randomized controlled trial. Am J Clin Nutr 2018;107(4):550-7. doi: 10.1093/ajcn/nqy019.

65. Kinsey AW, Eddy WR, Madzima TA, Panton LB, Arciero PJ, Kim JS, Ormsbee MJ. Influence of night-time protein and carbohydrate intake on appetite and cardiometabolic risk in sedentary overweight and obese women. Br J Nutr 2014;112(3):320-7. doi: 10.1017/s0007114514001068.

66. Kirk B, Mooney K, Amirabdollahian F, Khaiyat O. Exercise and Dietary-Protein as a Countermeasure to Skeletal Muscle Weakness: Liverpool Hope University - Sarcopenia Aging Trial (LHU-SAT). Front Physiol 2019;10:445. doi: 10.3389/fphys.2019.00445.

67. Kirk B, Mooney K, Vogrin S, Jackson M, Duque G, Khaiyat O, Amirabdollahian F. Leucine-enriched whey protein supplementation, resistance-based exercise, and cardiometabolic health in older adults: a randomized controlled trial. J Cachexia Sarcopenia Muscle 2021;12(6):2022-33. doi: 10.1002/jcsm.12805.

68. Kjølbæk L, Sørensen LB, Søndertoft NB, Rasmussen CK, Lorenzen JK, Serena A, Astrup A, Larsen LH. Protein supplements after weight loss do not improve weight maintenance compared with recommended dietary protein intake despite beneficial effects on appetite sensation and energy expenditure: a randomized, controlled, double-blinded trial. Am J Clin Nutr 2017;106(2):684-97. doi: 10.3945/ajcn.115.129528.

69. Larsen AE, Bibby BM, Hansen M. Effect of a Whey Protein Supplement on Preservation of Fat Free Mass in Overweight and Obese Individuals on an Energy Restricted Very Low Caloric Diet. Nutrients 2018;10(12). doi: 10.3390/nu10121918.

70. Laviolette L, Lands LC, Dauletbaev N, Saey D, Milot J, Provencher S, LeBlanc P, Maltais F. Combined effect of dietary supplementation with pressurized whey and exercise training in chronic obstructive pulmonary disease: a randomized, controlled, double-blind pilot study. J Med Food 2010;13(3):589-98. doi: 10.1089/jmf.2009.0142.

71. Lee YM, Skurk T, Hennig M, Hauner H. Effect of a milk drink supplemented with whey peptides on blood pressure in patients with mild hypertension. Eur J Nutr 2007;46(1):21-7. doi: 10.1007/s00394-006-0625-8.

72. Li C, Meng H, Wu S, Fang A, Liao G, Tan X, Chen P, Wang X, Chen S, Zhu H. Daily Supplementation With Whey, Soy, or Whey-Soy Blended Protein for 6 Months Maintained Lean Muscle Mass and Physical Performance in Older Adults With Low Lean Mass. J Acad Nutr Diet 2021;121(6):1035-48.e6. doi: 10.1016/j.jand.2021.01.006.

73. Lopes Gomes D, Moehlecke M, Lopes da Silva FB, Dutra ES, D'Agord Schaan B, Baiocchi de Carvalho KM. Whey Protein Supplementation Enhances Body Fat and Weight Loss in Women Long After Bariatric Surgery: a Randomized Controlled Trial. Obes Surg 2017;27(2):424-31. doi: 10.1007/s11695-016-2308-8.

74. Lynch HM, Buman MP, Dickinson JM, Ransdell LB, Johnston CS, Wharton CM. No Significant Differences in Muscle Growth and Strength Development When Consuming Soy and Whey Protein Supplements Matched for Leucine Following a 12 Week Resistance Training Program in Men and Women: A Randomized Trial. Int J Environ Res Public Health 2020;17(11). doi: 10.3390/ijerph17113871.

75. Ma J, Jesudason DR, Stevens JE, Keogh JB, Jones KL, Clifton PM, Horowitz M, Rayner CK. Sustained effects of a protein 'preload' on glycaemia and gastric emptying over 4 weeks in patients with type 2 diabetes: A randomized clinical trial. Diabetes Res Clin Pract 2015;108(2):e31-4. doi: 10.1016/j.diabres.2015.02.019.

76. Martens EA, Gonnissen HK, Gatta-Cherifi B, Janssens PL, Westerterp-Plantenga MS. Maintenance of energy expenditure on high-protein vs. high-carbohydrate diets at a constant body weight may prevent a positive energy balance. Clin Nutr 2015;34(5):968-75. doi: 10.1016/j.clnu.2014.10.007.

77. Matsuoka R, Kamachi K, Usuda M, Wang W, Masuda Y, Kunou M, Tanaka A, Utsunomiya K. Lactic-fermented egg white improves visceral fat obesity in Japanese subjects—double-blind, placebo-controlled study. Lipids in Health and Disease 2017;16(1):237. doi: 10.1186/s12944-017-0631-2.

78. McAdam JS, McGinnis KD, Beck DT, Haun CT, Romero MA, Mumford PW, Roberson PA, Young KC, Lohse KR, Lockwood CM, et al. Effect of Whey Protein Supplementation on Physical Performance and Body Composition in Army Initial Entry Training Soldiers. Nutrients 2018;10(9). doi: 10.3390/nu10091248.

79. Mobley CB, Haun CT, Roberson PA, Mumford PW, Romero MA, Kephart WC, Anderson RG, Vann CG, Osburn SC, Pledge CD, et al. Effects of Whey, Soy or Leucine Supplementation with 12 Weeks of Resistance Training on Strength, Body Composition, and Skeletal Muscle and Adipose Tissue Histological Attributes in College-Aged Males. Nutrients 2017;9(9). doi: 10.3390/nu9090972.

80. Moeller LE, Peterson CT, Hanson KB, Dent SB, Lewis DS, King DS, Alekel DL. Isoflavone-rich soy protein prevents loss of hip lean mass but does not prevent the shift in regional fat distribution in perimenopausal women. Menopause 2003;10(4):322-31. doi: 10.1097/01.Gme.0000054763.94658.Fd.

81. Mohammadi-Sartang M, Bellissimo N, Totosy de Zepetnek JO, Brett NR, Mazloomi SM, Fararouie M, Bedeltavana A, Famouri M, Mazloom Z. The effect of daily fortified yogurt consumption on weight loss in adults with metabolic syndrome: A 10-week randomized controlled trial. Nutr Metab Cardiovasc Dis 2018;28(6):565-74. doi: 10.1016/j.numecd.2018.03.001.

82. Mojtahedi MC, Thorpe MP, Karampinos DC, Johnson CL, Layman DK, Georgiadis JG, Evans EM. The effects of a higher protein intake during energy restriction on changes in body composition and physical function in older women. J Gerontol A Biol Sci Med Sci 2011;66(11):1218-25. doi: 10.1093/gerona/glr120.

83. Mori H, Tokuda Y. Effect of whey protein supplementation after resistance exercise on the muscle mass and physical function of healthy older women: A randomized controlled trial. Geriatr Gerontol Int 2018;18(9):1398-404. doi: 10.1111/ggi.13499.

84. Nabuco HCG, Tomeleri CM, Fernandes RR, Sugihara Junior P, Cavalcante EF, Cunha PM, Antunes M, Nunes JP, Venturini D, Barbosa DS, et al. Effect of whey protein supplementation combined with resistance training on body composition, muscular strength, functional capacity, and plasma-metabolism biomarkers in older women with sarcopenic obesity: A randomized, double-blind, placebo-controlled trial. Clin Nutr ESPEN 2019;32:88-95. doi: 10.1016/j.clnesp.2019.04.007.

85. Nabuco HCG, Tomeleri CM, Fernandes RR, Sugihara Junior P, Cavalcante EF, Venturini D, Barbosa DS, Silva AM, Sardinha LB, Cyrino ES. Effects of Protein Intake Beyond Habitual Intakes Associated With Resistance Training on Metabolic Syndrome-Related Parameters, Isokinetic Strength, and Body Composition in Older Women. J Aging Phys Act 2019;27(4):545–52. doi: 10.1123/japa.2018-0370.

86. Ormsbee MJ, Kinsey AW, Eddy WR, Madzima TA, Arciero PJ, Figueroa A, Panton LB. The influence of nighttime feeding of carbohydrate or protein combined with exercise training on appetite and cardiometabolic risk in young obese women. Appl Physiol Nutr Metab 2015;40(1):37-45. doi: 10.1139/apnm-2014-0256.

87. Padhi EM, Blewett HJ, Duncan AM, Guzman RP, Hawke A, Seetharaman K, Tsao R, Wolever TM, Ramdath DD. Whole Soy Flour Incorporated into a Muffin and Consumed at 2 Doses of Soy Protein Does Not Lower LDL Cholesterol in a Randomized, Double-Blind Controlled Trial of Hypercholesterolemic Adults. J Nutr 2015;145(12):2665-74. doi: 10.3945/jn.115.219873.

88. Pal S, Ellis V, Dhaliwal S. Effects of whey protein isolate on body composition, lipids, insulin and glucose in overweight and obese individuals. Br J Nutr 2010;104(5):716-23. doi: 10.1017/s0007114510000991.

89. Pal S, Ellis V. The chronic effects of whey proteins on blood pressure, vascular function, and inflammatory markers in overweight individuals. Obesity (Silver Spring) 2010;18(7):1354-9. doi: 10.1038/oby.2009.397.

90. Pal S, Ellis V, Ho S. Acute effects of whey protein isolate on cardiovascular risk factors in overweight, post-menopausal women. Atherosclerosis 2010;212(1):339-44. doi: 10.1016/j.atherosclerosis.2010.05.032.

91. Pal S, Radavelli-Bagatini S. The effects of whey protein on cardiometabolic risk factors. Obes Rev 2013;14(4):324-43. doi: 10.1111/obr.12005.

92. Petyaev IM, Dovgalevsky PY, Klochkov VA, Chalyk NE, Kyle N. Whey protein lycosome formulation improves vascular functions and plasma lipids with reduction of markers of inflammation and oxidative stress in prehypertension. ScientificWorldJournal 2012;2012:269476. doi: 10.1100/2012/269476.

93. Piccolo BD, Comerford KB, Karakas SE, Knotts TA, Fiehn O, Adams SH. Whey protein supplementation does not alter plasma branched-chained amino acid profiles but results in unique metabolomics patterns in obese women enrolled in an 8-week weight loss trial. J Nutr 2015;145(4):691-700. doi: 10.3945/jn.114.203943.

94. Pins JJ, Keenan JM. Effects of whey peptides on cardiovascular disease risk factors. J Clin Hypertens (Greenwich) 2006;8(11):775-82.

95. Rakvaag E, Fuglsang-Nielsen R, Bach Knudsen KE, Hermansen K, Gregersen S. The Combination of Whey Protein and Dietary Fiber Does Not Alter Low-Grade Inflammation or Adipose Tissue Gene Expression in Adults with Abdominal Obesity. Rev Diabet Stud 2019;15:83-94. doi: 10.1900/rds.2019.15.83.

96. Reidy PT, Borack MS, Markofski MM, Dickinson JM, Deer RR, Husaini SH, Walker DK, Igbinigie S, Robertson SM, Cope MB, et al. Protein Supplementation Has Minimal Effects on Muscle Adaptations during Resistance Exercise Training in Young Men: A Double-Blind Randomized Clinical Trial. J Nutr 2016;146(9):1660-9. doi: 10.3945/jn.116.231803.

97. Reidy PT, Fry CS, Igbinigie S, Deer RR, Jennings K, Cope MB, Mukherjea R, Volpi E, Rasmussen BB. Protein Supplementation Does Not Affect Myogenic Adaptations to Resistance Training. Med Sci Sports Exerc 2017;49(6):1197-208. doi: 10.1249/mss.0000000000001224.

98. Reimer RA, Willis HJ, Tunnicliffe JM, Park H, Madsen KL, Soto-Vaca A. Inulin-type fructans and whey protein both modulate appetite but only fructans alter gut microbiota in adults with overweight/obesity: A randomized controlled trial. Molecular Nutrition & Food Research 2017;61(11):1700484. doi: <https://doi.org/10.1002/mnfr.201700484>.

99. Roberson PA, Mobley CB, Romero MA, Haun CT, Osburn SC, Mumford PW, Vann CG, Greer RA, Ferrando AA, Roberts MD. LAT1 Protein Content Increases Following 12 Weeks of Resistance Exercise Training in Human Skeletal Muscle. Front Nutr 2020;7:628405. doi: 10.3389/fnut.2020.628405.

100. Rondanelli M, Klersy C, Terracol G, Talluri J, Maugeri R, Guido D, Faliva MA, Solerte BS, Fioravanti M, Lukaski H, et al. Whey protein, amino acids, and vitamin D supplementation with physical activity increases fat-free mass and strength, functionality, and quality of life and decreases inflammation in sarcopenic elderly. Am J Clin Nutr 2016;103(3):830-40. doi: 10.3945/ajcn.115.113357.

101. Sahathevan S, Se CH, Ng S, Khor BH, Chinna K, Goh BL, Gafor HA, Bavanandan S, Ahmad G, Karupaiah T. Clinical efficacy and feasibility of whey protein isolates supplementation in malnourished peritoneal dialysis patients: A multicenter, parallel, open-label randomized controlled trial. Clin Nutr ESPEN 2018;25:68-77. doi: 10.1016/j.clnesp.2018.04.002.

102. Sattler FR, Rajicic N, Mulligan K, Yarasheski KE, Koletar SL, Zolopa A, Alston Smith B, Zackin R, Bistrian B. Evaluation of high-protein supplementation in weight-stable HIV-positive subjects with a history of weight loss: a randomized, double-blind, multicenter trial. Am J Clin Nutr 2008;88(5):1313-21. doi: 10.3945/ajcn.2006.23583.

103. Sohrabi Z, Eftekhari MH, Eskandari MH, Rezaianzadeh A, Sagheb MM. Intradialytic Oral Protein Supplementation and Nutritional and Inflammation Outcomes in Hemodialysis: A Randomized Controlled Trial. Am J Kidney Dis 2016;68(1):122-30. doi: 10.1053/j.ajkd.2016.02.050.

104. Stragier S, Baudry S, Poortmans J, Duchateau J, Carpentier A. Leucine-enriched protein supplementation does not influence neuromuscular adaptations in response to a 6-month strength training programme in older adults. Exp Gerontol 2016;82:58-66. doi: 10.1016/j.exger.2016.06.002.

105. Stojkovic V, Simpson CA, Sullivan RR, Cusano AM, Kerstetter JE, Kenny AM, Insogna KL, Bihuniak JD. The Effect of Dietary Glycemic Properties on Markers of Inflammation, Insulin Resistance, and Body Composition in Postmenopausal American Women: An Ancillary Study from a Multicenter Protein Supplementation Trial. Nutrients 2017;9(5). doi: 10.3390/nu9050484.

106. Sugawara K, Takahashi H, Kashiwagura T, Yamada K, Yanagida S, Homma M, Dairiki K, Sasaki H, Kawagoshi A, Satake M, et al. Effect of anti-inflammatory supplementation with whey peptide and exercise therapy in patients with COPD. Respir Med 2012;106(11):1526-34. doi: 10.1016/j.rmed.2012.07.001.

107. Sukumar D, Ambia-Sobhan H, Zurfluh R, Schlussel Y, Stahl TJ, Gordon CL, Shapses SA. Areal and volumetric bone mineral density and geometry at two levels of protein intake during caloric restriction: a randomized, controlled trial. J Bone Miner Res 2011;26(6):1339-48. doi: 10.1002/jbmr.318.

108. Tahavorgar A, Vafa M, Shidfar F, Gohari M, Heydari I. Whey protein preloads are more beneficial than soy protein preloads in regulating appetite, calorie intake, anthropometry, and body composition of overweight and obese men. Nutr Res 2014;34(10):856-61. doi: 10.1016/j.nutres.2014.08.015.

109. Taylor LW, Wilborn C, Roberts MD, White A, Dugan K. Eight weeks of pre- and postexercise whey protein supplementation increases lean body mass and improves performance in Division III collegiate female basketball players. Appl Physiol Nutr Metab 2016;41(3):249-54. doi: 10.1139/apnm-2015-0463.

110. Tovar J, Nilsson A, Johansson M, Ekesbo R, Åberg A-M, Johansson U, Björck I. A diet based on multiple functional concepts improves cardiometabolic risk parameters in healthy subjects. Nutrition & Metabolism 2012;9(1):29. doi: 10.1186/1743-7075-9-29.

111. Tovar J, Johansson M, Björck I. A multifunctional diet improves cardiometabolic-related biomarkers independently of weight changes: an 8-week randomized controlled intervention in healthy overweight and obese subjects. Eur J Nutr 2016;55(7):2295-306. doi: 10.1007/s00394-015-1039-2.

112. Sheikholeslami Vatani D, Ahmadi Kani Golzar F. Changes in antioxidant status and cardiovascular risk factors of overweight young men after six weeks supplementation of whey protein isolate and resistance training. Appetite 2012;59(3):673-8. doi: 10.1016/j.appet.2012.08.005.

113. Verreijen AM, Verlaan S, Engberink MF, Swinkels S, de Vogel-van den Bosch J, Weijs PJ. A high whey protein-, leucine-, and vitamin D-enriched supplement preserves muscle mass during intentional weight loss in obese older adults: a double-blind randomized controlled trial. Am J Clin Nutr 2015;101(2):279-86. doi: 10.3945/ajcn.114.090290.

114. Verreijen AM, Engberink MF, Memelink RG, van der Plas SE, Visser M, Weijs PJ. Effect of a high protein diet and/or resistance exercise on the preservation of fat free mass during weight loss in overweight and obese older adults: a randomized controlled trial. Nutr J 2017;16(1):10. doi: 10.1186/s12937-017-0229-6.

115. Volek JS, Volk BM, Gómez AL, Kunces LJ, Kupchak BR, Freidenreich DJ, Aristizabal JC, Saenz C, Dunn-Lewis C, Ballard KD, et al. Whey protein supplementation during resistance training augments lean body mass. J Am Coll Nutr 2013;32(2):122-35. doi: 10.1080/07315724.2013.793580.

116. Watson LE, Phillips LK, Wu T, Bound MJ, Checklin HL, Grivell J, Jones KL, Clifton PM, Horowitz M, Rayner CK. A whey/guar "preload" improves postprandial glycaemia and glycated haemoglobin levels in type 2 diabetes: A 12-week, single-blind, randomized, placebo-controlled trial. Diabetes Obes Metab 2019;21(4):930-8. doi: 10.1111/dom.13604.

117. Weinheimer EM, Conley TB, Kobza VM, Sands LP, Lim E, Janle EM, Campbell WW. Whey protein supplementation does not affect exercise training-induced changes in body composition and indices of metabolic syndrome in middle-aged overweight and obese adults. J Nutr 2012;142(8):1532-9. doi: 10.3945/jn.111.153619.

118. Weisgarber KD, Candow DG, Farthing JP. Whey protein and high-volume resistance training in postmenopausal women. J Nutr Health Aging 2015;19(5):511-7. doi: 10.1007/s12603-015-0454-7.

119. Wu T, Little TJ, Bound MJ, Borg M, Zhang X, Deacon CF, Horowitz M, Jones KL, Rayner CK. A Protein Preload Enhances the Glucose-Lowering Efficacy of Vildagliptin in Type 2 Diabetes. Diabetes Care 2016;39(4):511-7. doi: 10.2337/dc15-2298.

120. Yang J, Wang HP, Tong X, Li ZN, Xu JY, Zhou L, Zhou BY, Qin LQ. Effect of whey protein on blood pressure in pre- and mildly hypertensive adults: A randomized controlled study. Food Sci Nutr 2019;7(5):1857-64. doi: 10.1002/fsn3.1040.

121. Zhu K, Kerr DA, Meng X, Devine A, Solah V, Binns CW, Prince RL. Two-Year Whey Protein Supplementation Did Not Enhance Muscle Mass and Physical Function in Well-Nourished Healthy Older Postmenopausal Women. J Nutr 2015;145(11):2520-6. doi: 10.3945/jn.115.218297.
